# Metabolomics Investigation of an Association of Induced Features and Corresponding Fungus during the Co-culture of *Trametes versicolor* and *Ganoderma applanatum*

**DOI:** 10.3389/fmicb.2017.02647

**Published:** 2018-01-09

**Authors:** Xiao-Yan Xu, Xiao-Ting Shen, Xiao-Jie Yuan, Yuan-Ming Zhou, Huan Fan, Li-Ping Zhu, Feng-Yu Du, Martin Sadilek, Jie Yang, Bin Qiao, Song Yang

**Affiliations:** ^1^Shandong Province Key Laboratory of Applied Mycology, Qingdao International Center on Microbes Utilizing Biogas, School of Life Science, Qingdao Agricultural University, Qingdao, China; ^2^Central Laboratory, Qingdao Agricultural University, Qingdao, China; ^3^Tianjin Animal Science and Veterinary Research Institute, Tianjin Academy of Agricultural Sciences, Tianjin, China; ^4^School of Chemistry and Pharmacy, Qingdao Agricultural University, Qingdao, China; ^5^Department of Chemistry, University of Washington, Seattle, WA, United States; ^6^Department of Biochemistry and Molecular Biology, School of Basic Medical Sciences, Tianjin Medical University, Tianjin, China; ^7^School of Chemical Engineering and Technology, Tianjin University, Tianjin, China; ^8^Key Laboratory of Systems Bioengineering, Ministry of Education, Tianjin University, Tianjin, China

**Keywords:** basidiomycete, interaction, ^13^C-labeling, origin of induced features, phenyl polyketide, metabolite identification, LC-MS

## Abstract

The co-culture of *Trametes versicolor* and *Ganoderma applanatum* is a model of intense basidiomycete interaction, which induces many newly synthesized or highly produced features. Currently, one of the major challenges is an identification of the origin of induced features during the co-culture. Herein, we report a ^13^C-dynamic labeling analysis used to determine an association of induced features and corresponding fungus even if the identities of metabolites were not available or almost nothing was known of biochemical aspects. After the co-culture of *T. versicolor* and *G. applanatum* for 10 days, the mycelium pellets of *T. versicolor* and *G. applanatum* were sterilely harvested and then mono-cultured in the liquid medium containing half fresh medium with ^13^C-labeled glucose as carbon source and half co-cultured supernatants collected on day 10. ^13^C-labeled metabolome analyzed by LC-MS revealed that 31 induced features including 3-phenyllactic acid and orsellinic acid were isotopically labeled in the mono-culture after the co-culture stimulation. Twenty features were derived from *T. versicolor*, 6 from *G. applanatum*, and 5 features were synthesized by both *T. versicolor* and *G. applanatum*. ^13^C-labeling further suggested that 12 features such as previously identified novel xyloside [N-(4-methoxyphenyl)formamide 2-O-beta-D-xyloside] were likely induced through the direct physical interaction of mycelia. Use of molecular network analysis combined with ^13^C-labeling provided an insight into the link between the generation of structural analogs and producing fungus. Compound **1** with m/z 309.0757, increased 15.4-fold in the co-culture and observed ^13^C incorporation in the mono-culture of both *T. versicolor* and *G. applanatum*, was purified and identified as a phenyl polyketide, 2,5,6-trihydroxy-4, 6-diphenylcyclohex-4-ene-1,3-dione. The biological activity study indicated that this compound has a potential to inhibit cell viability of leukemic cell line U937. The current work sets an important basis for further investigations including novel metabolites discovery and biosynthetic capacity improvement.

## Introduction

Secondary metabolites are an important source of valuable drug leads, of which compounds derived from various fungi, especially medicinal fungi of basidiomycetes, represent an important part. To explore chemical diversity, several approaches such as epigenetic modification or non-targeted metabolic pathway manipulation have recently been developed in the *Aspergillus* and *Streptomyces* species (Scherlach and Hertweck, 2009; Chiang et al., 2011). In the case of basidiomycetes, the induction of novel secondary metabolites or enhancement of secreting extracellular enzymes can be achieved by activating cryptic biosynthetic pathways through establishment of a fungi interaction in the co-culture (Peiris et al., 2008; Hiscox et al., 2010). This co-culture strategy mimics natural ecosystem, in which interspecies interaction of basidiomycetes is very common (Hiscox et al., 2015, 2017). Recently, Zheng et al. demonstrated that co-culture of basidiomycetes *Inonotus obliquus* and *Phellinus punctatus* resulted in the accumulation of lanostane-type triterpenoids, polyphenols, and melanins, compounds capable of scavenging free radicals and inhibiting tumor cell proliferation (Zheng et al., 2011). The co-culture of *Trichoderma Reesei* with *Coprinus comatus* was an important approach for an on-site generation of lignocellulolytic enzyme leading to the increase of lignocellulose degradation rate as described by Ma and Ruan (2015). In our previous work, fifteen wood-decaying basidiomycetes and two straw-decaying basidiomycetes were used to establish 136 pairwise co-cultures on agar plates (Yao et al., 2016). The co-culture system of *Trametes versicolor* and *Ganoderma applanatum* showed an interaction zone, in which the accumulation of a series of known carboxylic acids as well as novel xylosides were observed.

The application of co-culture has enhanced the number of discovered novel secondary metabolites but also raises several challenges. One of the major challenges is how to timely and accurately identify the species responsible for the newly induced metabolites during the microorganism interaction. Typically, the structure of compounds and related biochemical information were required to verify the unique secondary metabolite and its particular producer (Riedlinger et al., 2006). Recently, a combined technique of nanospray desorption electrospray ionization and matrix-assisted laser desorption/ionization-time of flight mass spectrometry has been developed as a platform to reveal many unknown metabolites produced by *Streptomyces coelicolor* when paired with five other actinomycetes (Traxler et al., 2013). However, this approach was limited to associate the novel metabolites with corresponding producer grown on agar plates and was not suitable to the co-culture in the liquid medium. The metabolic incorporation of stable isotopes ^13^C or ^15^N is a powerful approach used for quantitative proteome and metabolome studies (Julka and Regnier, 2004; Yang et al., 2010). Moreover, ^13^C-dynamic labeling analysis has been applied for analyzing the metabolite turnover rates, distinguishing the flux distribution between two pathways starting from the same metabolic point, and interpreting the synthetic process of novel metabolites (Yang et al., 2012; Shlomi et al., 2014; Hammerl et al., 2017). With the advantage of a ^13^C-labeling approach, the current study reports on the identification of the producer of novel induced metabolites in liquid medium after the two interactive mycelia were sterilely separated and mono-cultured after the induction by the co-culture.

The research of the interspecies crosstalk expands our possibilities to discover novel metabolites, and also increases our understanding of how these metabolites are induced in microbial consortia. A chemical warfare in the fungal-fungal communication is often described as diffusion of harmful and chemically complex metabolites from one partner to the other (Bertrand et al., 2014). For instance, the interaction between the *Aspergillus niger* and *Aspergillus flavus* led to the inhibition of aflatoxin B1 produced by *A. flavus* through signal molecules downregulating expression of major biosynthetic genes (Xing et al., 2017). Under some conditions, co-cultivation appears to trigger production and accumulation of novel metabolites without the involvement of released signaling molecules. For instance, co-cultivation with *Corynebacteria glutamicum* or *Tsukamurella pulmonis* was indicated to stimulate a novel pathway in *S. endus*, contributing to a new heterocyclic chromophore-containing antibiotics alchivemycin A (Onaka et al., 2011). Notably, the mono-culture of *S. endus* did not generate the same compound with or without the addition of filter sterilized supernatants from bacterial culture. The production of alchivemycin A therefore appeared to require a direct physical interaction between *S. endus* and the coryneform bacteria cells. For this research, ^13^C dynamic labeling was further utilized to suggest the potential mechanism of the induction of increased and newly synthesized features.

In this work, we built upon the characteristic of mycelial pellets of basidiomycetes and ^13^C-labeling analysis, to analyze and catalog a broad range of ^13^C-labeled features, which were highly accumulated in the co-culture of *T. versicolor* and *G. applanatum*. ^13^C dynamic labeling further suggested two potential mechanism of the induction of these features. Ultimately, compound **1** with M+H^+^ m/z 309.0757, that displayed ^13^C incorporation in the mono-culture of both *T. versicolor* and *G. applanatum*, was isolated and identified as novel phenyl polyketide, and its biological activity was evaluated.

## Materials and methods

### Chemicals

All chemicals including standards (ascorbic acid and phenolic antioxidant 2,6-di-*tert*-butyl-4-methoxyphenol) were purchased from either Sigma-Aldrich (St. Louis, MO, USA) or TCI (Kita-ku, Tokyo, Japan). Millipore water (Billerica, MA, USA) was used for the preparation of all the media and sample solutions.

### Fungi material and culture conditions

*G. applanatum* (CGMCC No. 5.249) and *T. versicolor* (CGMCC No. 12241) were deposited at the Shandong Province Key Lab of Applied Mycology in China. The culture medium was supplied at concentrations as follows: 2 g glucose, 0.2 g KH_2_PO_4_, 0.1 g MgSO_4_, 0.4 g peptone, and 4 g agar (only in solid medium) in 200 mL of sterilized water.

### Mono-culture of *T. versicolor* and *G. applanatum* on agar plate

The mono-culture procedure was adapted from our previous publication (Yao et al., 2016), and mono-culture medium was the same as above. Briefly, a 5 mm agar plug of each fungus scraped from agar slant culture-medium was cultured on a Petri dish (9 cm diameter), and were incubated at 28°C for 10 days.

### Co-culture of *T. versicolor* and *G. applanatum* in liquid medium and sample preparation

Eight 5 mm agar plugs of *T. versicolor* and *G. applanatum* were separately pre-cultured in 500 mL shake flask containing 200 mL of culture medium at 28°C for 4 days on orbital shakers at 180 rpm. Then 100 mL culture broth of *G. applanatum* was transferred into the culture of 100 mL *T. versicolor*, and co-cultivated up to 18 days. All the co-cultures had three independent biological replicates. At the harvest time, 10 mL of co -culture broth was filtered by using MILLEX-GP PES membrane filters (0.22 μm, 33 mm, Merck Millipore, Germany) and the filtrate was dried in a freeze-dryer ALPHA 1-2LDplus (Christ, Osterode, Germany). Five milliliters of freshly prepared dichloromethane/methanol/water (64:36:8, v/v) solvent mixture was added to the dried samples (Yao et al., 2016). The sample extractions were carried out in a water bath sonicator (KQ-300GVDV, Kunshan, China) at 25°C for 20 min, and were centrifuged at 12,000 rpm for 10 min. Finally, the extracts were dried on a rotational vacuum concentrator (Christ, Osterode, Germany) and stored in a −80°C freezer.

### Measurement of the metabolome

The extracts were dissolved in 200 μL methanol, and then were centrifuged at 12,000 rpm for 15 min. The supernatants were transferred into 250 μL Agilent autosampler vials. The samples were analyzed on an Agilent liquid chromatograph-quadrupole time-of-flight mass spectrometer (LC-QTOF-MS, Agilent 1290 Infinity-6530B, Agilent Technologies, Santa Clara, CA, USA) as previously described (Cui et al., 2016; Hu et al., 2016). Briefly, 10 μL of the samples was separated on an Acquity UPLC BEH C18 column (100 × 2.1 mm, 1.7 μm, Waters, Milford, MA, USA). The mobile phase A was water with 0.1% formic acid. The mobile phase B was pure acetonitrile. The reversed-phase liquid chromatographic elution gradient was optimized in order to maximize the resolution of the induced features. The gradient was the following: 0–3 min, 5% B, 3–12 min, 5–40% B, 12–38 min, 40–95% B, 38–46 min, 95% B, 46–48 min, 95–5% B, 48–55 min, 5% B. This shallow gradient provided reproducible separation for many co-eluting compounds in a time window from 5.0 to 8.0 min. The TOF m/z range was set to 50–1,200 amu in centroid mode with a scan rate of 1.5 spectra/s. All the samples had three independent biological replicates. Each biological replicate had two analytical replicates.

### Data pre-processing and principal component analysis

LC-QTOF-MS data were converted into mzML format using MS Convert software (Holman et al., 2014). Data pre-processing and statistical analysis were performed with MZmine 2 (Version 2.11) (Pluskal et al., 2010) and SIMCA-P 11.5. For the MZmine 2, the peak detection threshold for MS signal intensity was set to 1.0 × 10^3^. The chromatogram building was realized using a minimum time span of 0.01 min, minimum height of 2.5 × 10^3^, and m/z tolerance of 0.005 (or 10 ppm). Chromatograms were deconvoluted with the following settings: search minimum in absolute retention time (RT) range 0.1 min, minimum relative height 10%, minimum absolute height 2.5 × 10^3^ and baseline level 1.2. The chromatogram isotopic peaks grouper algorithm was set as m/z tolerance of 0.005 (or 10 ppm) and absolute RT tolerance of 0.10 min. Chromatograms were peak aligned with m/z tolerance at 0.008 (or 15 ppm) and absolute RT tolerance 1 min. The peak list was eventually gap-filled with m/z tolerance at 0.008 (or 15 ppm), and absolute RT tolerance of 0.20 min. To classify m/z in the peak list, principal component analysis (PCA) was carried out by using SIMCA-P (version 11.5). Two steps were required to perform PCA. The first step was to set the variables m/z and RT as Primary ID and Secondary ID. The secondary step was to do the normalization of the variables with Pareto scaling. This normalized method was embedded in SIMPCA-P, which did not require further parameter tuning. After that, PCA were displayed by a scores plot, mainly observing the overall cluster of the different treatments as well as the presence of outliers. The correlation coefficient loading plot was used to identify the variables responsible for the clustering or separation of the treatments.

### Molecular network analysis

MS/MS data for molecular network analysis were acquired in targeted MS/MS mode on the same LC-QTOF-MS system (the precursor ions are listed in Table 1). The collision energy and m/z range for different precursor ions were optimized based on their own characteristics as our previous publication (Yao et al., 2016). MS/MS data were converted to mzML format, and then were subjected to the Molecular Networking workflow of Global Natural Products Social Molecular (GNPS at gnps.ucsd.edu) using the Group Mapping feature (Watrous et al., 2012; Wang et al., 2016). The subnetworks were generated with settings of minimum pairs cosine 0.65, parent mass tolerance 1.0 Dalton, ion tolerance 0.5 Dalton, maximum connected components 50, minimum matched peaks 6, minimum cluster size 2. The results were then visualized using Cytoscape (Version 3.1.1) (Su et al., 2014).

**Table 1:** List of induced features in the co-culture of *T. versicolor* and *G. applanatum* analyzed by LC-MS in the positive/negative mode and corresponding production fungus.

**m/z(–)**	**RT /min**	**Predictive elemental composition**	**Difference**	**Produced by fungus**	**^12^C signal intensity in the co-culture**	**Features discovered with the current, optimized chromatography**
136.0403	3.91	C_7_H_7_NO_2_	Newly synthesized	*G. applanatum*	4.00E+04	
165.0554	9.79	C_9_H_10_O_3_	Fold increase 15.8 ± 0.7	*T. versicolor and G. applanatum*	2.50E+04	
166.0507	8.65	C_8_H_9_NO_3_	Newly synthesized	NA	6.00E+04	
167.0348	9.90	C_8_H_8_O_4_	Fold increase 5.8 ± 0.6	*G. applanatum*	5.00E+03	
181.0615	6.46	C_8_H_10_N_2_O_3_	Newly synthesized	*T. versicolor*	4.00E+03	
195.0505	8.37	C_6_H_12_O_4_	Fold increase 25.3 ± 1.2	NA	ND	
244.0612	6.86	C_13_H_11_NO_4_	Newly synthesized	NA	7.00E+03	
249.1497	16.33	C_15_H_22_O_3_	Newly synthesized	*T. versicolor*	1.50E+04	+
251.0712	15.72	C_16_H_12_O_3_	Fold increase 7.3 ± 1.3	*T. versicolor*	6.00E+03	
269.0577	11.75	C_14_H_10_N_2_O_4_	Newly synthesized	*G. applanatum*	9.00E+03	+
271.0716	7.03	C_14_H_12_N_2_O_4_	Newly synthesized	*G. applanatum*	1.00E+05	
271.0725	10.25	C_14_H_12_N_2_O_4_	Newly synthesized	*G. applanatum*	7.00E+04	
279.0656	13.25	C_17_H_12_O_4_	Fold increase 5.2 ± 1.3	*T. versicolor*	2.50E+05	+
281.0808	11.93	C_17_H_14_O_4_	Fold increase 102.2 ± 4.8	*T. versicolor*	5.00E+04	
298.0928	8.65	C_13_H_17_NO_7_	Newly synthesized	NA	2.30E+04	
304.0626	3.33	C_9_H_13_N_3_O_9_	Newly synthesized	NA	ND	
306.0775	10.46	C_18_H_13_NO_4_	Newly synthesized	NA	ND	+
309.0756	12.04	C_18_H_14_O_5_	Fold increase 15.4 ± 1.2	*T. versicolor* and *G. applanatum*	2.50E+05	+
311.0931	11.49	C_18_H_16_O_5_	Fold increase 400 ± 7.1	*T. versicolor*	4.00E+04	
314.0899	3.32	C_14_H_13_N_5_O_4_	Newly synthesized	NA	ND	
330.2656	14.03	C_18_H_37_NO_4_	Fold increase 59.7 ± 8.1	*T. versicolor*	6.00E+04	
334.0733	11.44	C_20_H_9_N_5_O_4_	Newly synthesized	NA	ND	
337.0712	10.59	C_20_H_10_N_4_O_2_	Newly synthesized	NA	ND	+
341.1046	16.16	C_20_H_14_N_4_O_2_	Newly synthesized	*T. versicolor*	5.00E+03	+
342.2654	15.17	C_19_H_37_NO_4_	Newly synthesized	*T. versicolor* and *G. applanatum*	6.00E+04	
387.1926	20.12	C_19_H_36_N_2_S_3_	Newly synthesized	NA	ND	
389.2072	20.07	C_19_H_38_N_2_S_3_	Newly synthesized	NA	ND	
397.223	19.42	C_21_H_34_O_7_	Newly synthesized	NA	1.25E+04	
398.1058	13.53	C_25_H_13_N_5_O	Newly synthesized	NA	ND	+
399.2386	20.07	C_21_H_36_O_7_	Fold increase 103.3 ± 4.2	NA	3.00E+03	
405.0737	12.83	C_22_H_10_N_6_O_3_	Newly synthesized	NA	ND	
406.1043	7.89	C_22_H_13_N_7_O_2_	Newly synthesized	NA	ND	
415.2338	10.40	C_21_H_36_O_8_	Newly synthesized	NA	ND	
436.2698	15.86	C_24_H_39_NO_6_	Newly synthesized	*T. versicolor*	2.50E+04	+
454.2817	15.86	C_24_H_41_NO_7_	Newly synthesized	*T. versicolor*	1.50E+04	+
455.1685	8.64	C_21_H_24_N_6_O_6_	Newly synthesized	NA	ND	
472.2923	15.86	C_24_H_43_NO_8_	Newly synthesized	*T. versicolor*	1.25E+04	+
490.3025	15.06	C_24_H_45_NO_9_	Newly synthesized	*T. versicolor*	3.40E+04	+
567.3139	15.87	C_25_H_48_N_2_O_12_	Newly synthesized	*T. versicolor*	7.00E+03	+
581.1208	13.24	C_36_H_22_O_8_	Newly synthesized	NA	ND	+
586.2857	16.44	C_28_H_45_NO_12_	Newly synthesized	NA	2.00E+03	+
597.1968	8.63	C_31_H_34_O_12_	Newly synthesized	NA	ND	
629.1419	11.77	C_30_H_22_N_4_O_12_	Newly synthesized	NA	ND	+
641.1461	12.65	C_19_H_29_N_7_O_18_	Newly synthesized	NA	ND	
138.0555	3.93	C_7_H_7_NO_2_	Newly synthesized	*G. applanatum*	8.00E+04	
140.0708	8.64	C_7_H_9_NO_2_	Fold increase 14.9 ± 1.3	*G. applanatum*	1.10E+04	
150.0548	8.53	C_8_H_7_NO_2_	Newly synthesized	NA	1.00E+05	
160.0377	3.84	C_4_H_5_N_3_O_4_	Newly synthesized	NA	ND	
168.0653	8.65	C_8_H_9_NO_3_	Newly synthesized	NA	8.00E+04	+
196.0944	9.16	C_10_H_13_NO_3_	Newly synthesized	NA	2.00E+03	+
216.1021	14.2	C_13_H_14_NO_2_	Newly synthesized	NA	1.00E+04	+
230.1177	19.07	C_14_H_15_NO_2_	Newly synthesized	NA	2.60E+03	
257.0919	9.18	C_14_H_12_N_2_O_3_	Newly synthesized	NA	ND	+
265.0860	11.92	C_17_H_12_O_3_	Fold increase 802.4 ± 9.6	NA	ND	+
268.2277	15.96	C_16_H_29_NO_2_	Newly synthesized	NA	4.00E+03	+
270.0979	3.27	C_13_H_11_N_5_O_2_	Newly synthesized	NA	ND	
272.1129	3.58	C_12_H_17_NO_6_	Newly synthesized	NA	ND	
273.0871	10.22	C_14_H_12_N_2_O_4_	Newly synthesized	*G. applanatum*	1.10E+05	
286.1436	14.85	C_17_H_19_NO_3_	Newly synthesized	NA	ND	
287.1030	10.61	C_16_H_10_N_6_	Newly synthesized	NA	ND	
288.2900	20.88	C_17_H_37_NO_2_	Fold increase 50.5 ± 3.3	*T. versicolor*	1.90E+04	+
298.2744	18.56	C_18_H_35_NO_2_	Newly synthesized	*T. versicolor*	4.00E+04	+
300.1076	8.65	C_13_H_17_NO_7_	Newly synthesized	*NA*	3.00E+04	
302.3069	21.38	C_18_H_39_NO_2_	Fold increase 24.4 ± 3.1	*T. versicolor and G. applanatum*	4.80E+05	
322.0898	8.7	C_11_H_11_N_7_O_5_	Newly synthesized	NA	ND	+
330.3371	26.77	C_20_H_43_NO_2_	Fold increase 152.3 ± 1.6	*T. versicolor*	5.60E+03	
332.2800	14.05	C_18_H_37_NO_4_	Fold increase 74.3 ± 4.6	*T. versicolor*	3.60E+04	
334.2953	14.95	C_18_H_39_NO_4_	Newly synthesized	*T. versicolor*	1.60E+04	
344.2803	15.17	C_19_H_37_NO_4_	Newly synthesized	*T. versicolor and G. applanatum*	1.25E+05	
348.1430	11.71	C_20_H_17_N_3_O_3_	Newly synthesized	NA	ND	
358.2955	15.66	C_20_H_39_NO_4_	Fold increase 25.2 ± 2.3	*T. versicolor*	3.00E+04	+
369.2269	13.47	C_16_H_28_N_6_O_4_	Newly synthesized	NA	ND	
372.3113	19.96	C_21_H_41_NO_4_	Newly synthesized	*T. versicolor and G. applanatum*	3.60E+05	
377.2302	20.09	C_18_H_28_N_6_O_3_	Newly synthesized	NA	ND	+
400.1019	7.96	C_17_H_9_N_11_O_2_	Newly synthesized	NA	ND	
432.1504	8.68	C_19_H_21_N_5_O_7_	Newly synthesized	NA	ND	
474.3068	15.86	C_24_H_43_NO_8_	Newly synthesized	*T. versicolor*	1.25E+04	+
492.3176	15.04	C_24_H_45_NO_9_	Newly synthesized	*T. versicolor*	1.50E+04	+
496.2886	16.43	C_15_H_41_N_2_O_4_	Newly synthesized	*T. versicolor*	4.00E+04	+
506.3332	15.85	C_25_H_47_NO_9_	Newly synthesized	*T. versicolor*	3.00E+04	+
599.2043	8.63	C_31_H_34_O_12_	Newly synthesized	NA	ND	
716.5259	18.91	C_42_H_69_NO_8_	Newly synthesized	NA	ND	

*Yellow label, the features were observed by LC-MS in both negative and positive modes. These features were only counted once. Plus mark, the features discovered in this work were tagged with plus mark. NA, the features had no ^13^C incorporation. ND, the features had weak MS signal intensity or no signal in the supernatants from the co-culture on day 10. Fold increase, fold value was the ratio of MS signal intensity of increased feature in the co-culture to that in the control mono-culture. Data show the mean with standard deviation calculated from three independent biological replicates*.

### ^13^C-labeling analysis

*T. versicolor* and *G. applanatum* were co-cultivated up to 10 days as described above. Then mycelium pellets of *T. versicolor* and *G. applanatum* were respectively harvested using sterilized tweezers based on the difference of diameter size and color. The mycelium pellets of *T. versicolor* had the size ranging from 6 to 8 mm with the color of faint yellow and those of *G. applanatum* had the size ranging from 2 to 4 mm with red color. The harvested mycelium pellets were washed with sterile water three times and then mono-cultured in 50 ml shake flask containing 10 mL fresh medium with ^13^C-labeled glucose at the final concentration of 5 g L^−1^ and 10 mL of co-cultured supernatants collected on day 10. For the control experiment no co-culture supernatants were added, only 20 mL of fresh medium with ^13^C-labeled glucose as carbon source. After the mono-culture for 10 or 20 days, 10 mL of *T. versicolor* and *G. applanatum* culture broths were harvested for analysis of ^13^C-labeling. All samples had three independent biological replicates and two analytical replicates. Samples were extracted and analyzed by LC-QTOF-MS as described above. The mass isotopomer distributions were corrected for the contribution from natural isotopes by a matrix-based method (Jennings and Matthews, 2005). The total ^13^C-incorporation for each feature was obtained by normalizing to its total carbon number as in our previous publication (Yang et al., 2013). Relative isotopic abundance (*M*i) for a feature in which *i* ^13^C atoms were incorporated was calculated by the Equation (1):

(1) $$\begin{array}{r} \text{M}i\left(\%\right)=\frac{m_{i}}{\sum_{j=0}^{n}m_{j}} \end{array}$$

where *m*_*i*_ represents the isotopic abundance for a feature in which *i* ^13^C atoms were incorporated and *n* represents the maximum number of ^13^C atoms incorporated.

Total ^13^C-incorporation of a feature with N carbon atoms was obtained by normalizing to its total carbon number according to the Equation (2):

(2) $$\begin{array}{r} \text{Total}{\;}^{13}\text{C}-\text{incorporation }\left(\%\right)=\frac{\sum_{i=1}^{\text{N}}i\times \text{M}i}{\text{N}} \end{array}$$

The significance of difference of ^13^C-incorporation between experimental data points was determined by *t*-tests (Origin 8.0). A *P*-value <0.05 was considered to be statistically significant.

### Isolation and purification of compound 1

Twenty liters of the co-cultured supernatant was extracted three times with ethyl acetate (EtOAc). 2.5 g of crude EtOAc extract was concentrated under reduced pressure and purified on a silica gel (200–300 mesh) column and then eluted with petroleum ether/ethyl acetate and chloroform/methanol system to yield ten fractions (Song et al., 2014). Eight hundred and fifty milligrams of fraction 6 and 7 from the chloroform/methanol elution (20:1 and 10:1, v/v) was used to a medium pressure liquid chromatography (Flash CO140080-0, Agela Technologies, China) and eluted with methanol/water (the elution gradient was 10–90% methanol in 65 min) to generate a mixture of 216 mg. This mixture was purified on a silica gel (200–300 mesh, Qingdao Haiyang Chem. Ind. Co. Ltd. China) column by eluting with petroleum ether/ethyl acetate/ methanol (3:3:1, v/v) and then separated on a preparative column (Venusil XBP C18, Agela Technologies, China) by eluting with methanol/water (the elution gradient was 10–50% methanol in 40 min and flow rate was 8 mL/min) to obtain 10 mg of compound **1**.

### NMR analysis of purified compound 1

^1^H, ^13^C, and 2D NMR spectra of the purified compound **1** were all performed by using a Bruker Avance 600 MHz spectrometer (Karlsruhe, Germany) at 25°C. The compound **1** was accurately weighted, and dissolved in 0.5 mL of deuterated methanol as the internal lock. The resulting spectra were manually phased and baseline corrected and calibrated to methanol, using TOPSPIN (Version 2.1, Bruker).

### Cell viability assay

The human leukemic cell line U937 and lung cancer cell line A549 were purchased from American Type Culture Collection (Manassas, VA, USA). They were cultured in RPMI-1640 (HyClone, USA) with 10% FBS (fetal bovine serum, Gibco). Cells were inoculated in 96-well plates at a density of 3,000 cells per well overnight and then treated to different concentrations of compound **1** for 48 h. The compound **1** was solved in ethanol. At a concentration of 300 μM of compound **1**, the ethanol was 0.8% (v/v) in the cell culture medium, showing minimal effect on the viability of both cell lines. The effect of compound **1** on the viability of U937 and A549 cells was evaluated with CellTiter 96®AQueous One Solution (Promega, Madison, WI, USA) (Soman et al., 2009). The absorbance was read at 490 nm with a microplate spectrophotometer (Multiskan FC, Thermo scientific, USA). Three independent experiments were performed and each one had six replicates. Data show the mean with error bar indicating standard deviation calculated from biological replicates by Origin 8.0. IC_50_ defined as the concentration with the inhibition of 50% cells was calculated by using SPSS (Statistical Package for Social Sciences) package 6, version 15.0.

### Antioxidant activity assay

The 2,2′-azinobis-(3-ethylbenzothiazoline-6-sulfonic acid) (ABTS) method used is based on the reduction of the ABTS^•+^ radical action by the antioxidants present in the sample (Rigano et al., 2014). Three biological replicates were performed. A solution of 7.4 mM ABTS^•+^ (5 mL) mixed with 140 mM K_2_S_2_O_8_ (88 μL) was prepared and stabilized for 12 h at 4°C in the dark. This mixture was then diluted by mixing ABTS^•+^ solution with ethanol (1:88) to obtain an absorbance of 0.70 ± 0.10 unit at 734 nm using a spectrophotometer (Multiskan FC, Thermo scientific, USA). Compound **1** (100 μL) and the standard controls (ascorbic acid and phenolic antioxidant 2,6-di-*tert*-butyl-4-methoxyphenol) reacted with 1 mL of diluted ABTS^•+^ solution for 2.5 min, and then the absorbance was taken at 734 nm against a blank constituted by ABTS^•+^ solution added with 100 μL of ethanol. ABTS^•+^ scavenging activity was calculated by the Equation (3):

(3) $$\begin{array}{r} \text{Scavenging effect }\left(\text{\%}\right)=\left(1-\frac{Abs.sample}{Abs.blank}\right)\times 100 \end{array}$$

where Abs blank = 100 μL of ethanol+1 mL of diluted ABTS^•+^ solution.

## Results

### Induced feature discovery in fungal interaction

**Figure 1:**
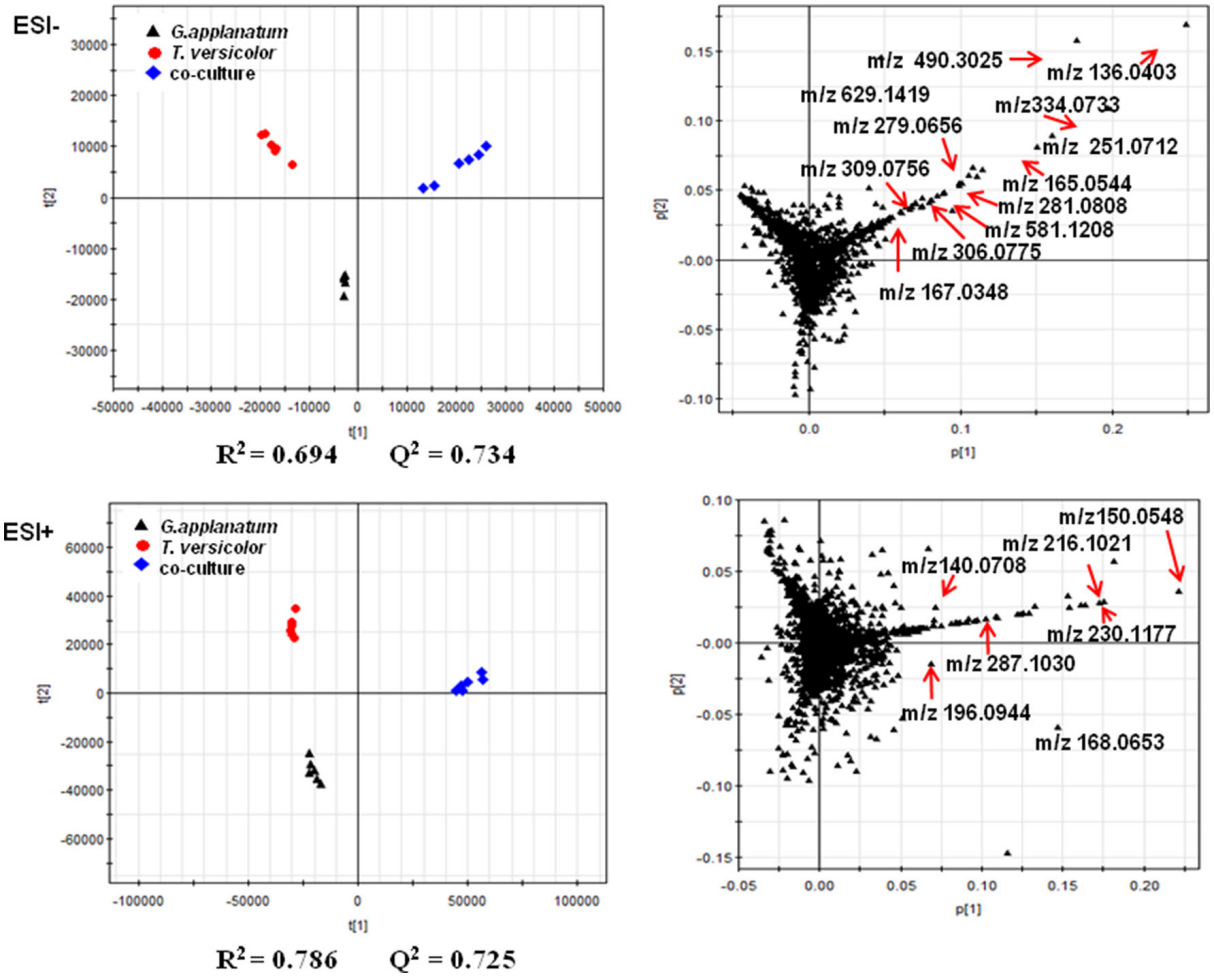
PCA of metabolomics data of co-culture and their corresponding mono-cultures on day 18 is shown. The score and loading plots of the data analyzed by LC-QTOF-MS in the negative mode (2287 features) and positive mode (2076 features), respectively. The parameters (*R*^2^ and *Q*^2^) of the score plots demonstrated the discriminative ability of this model. The scattered dots labeled with m/z were representative features mentioned in the Results section.

**Figure 2:**
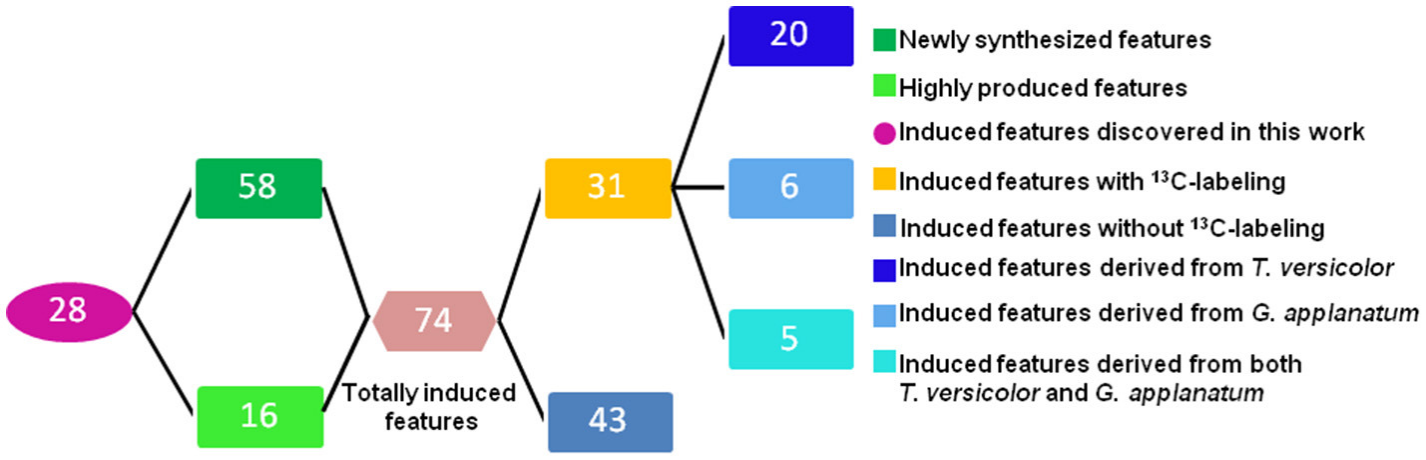
Overview of the number of the induced features in the co-culture of *T. versicolor* and *G. applanatum*.

Unsupervised PCA is well-suited for comparing different biological samples and identifying statistically significant differences (Chen et al., 2007). As shown in Figure 1 (left figure), examination of the scores plot showed that the co-culture treatment is clearly separated from the two mono-cultures. The variable features responsible for discriminating these three groups are shown in the loading plots (Figure 1, right). More than 4,000 features were recorded, out of which 58 features were detected only in the co-culture and 16 features were at least 5-fold more abundant in the co-culture than those in the control mono-culture (Table 1, Figure 2). Compared to our previous work (Yao et al., 2016), 28 additional features were discovered mainly due to the optimized chromatography (Table 1, Figure 2).

### ^13^C-labeling analysis to associate induced features with corresponding fungus

**Figure 3:**
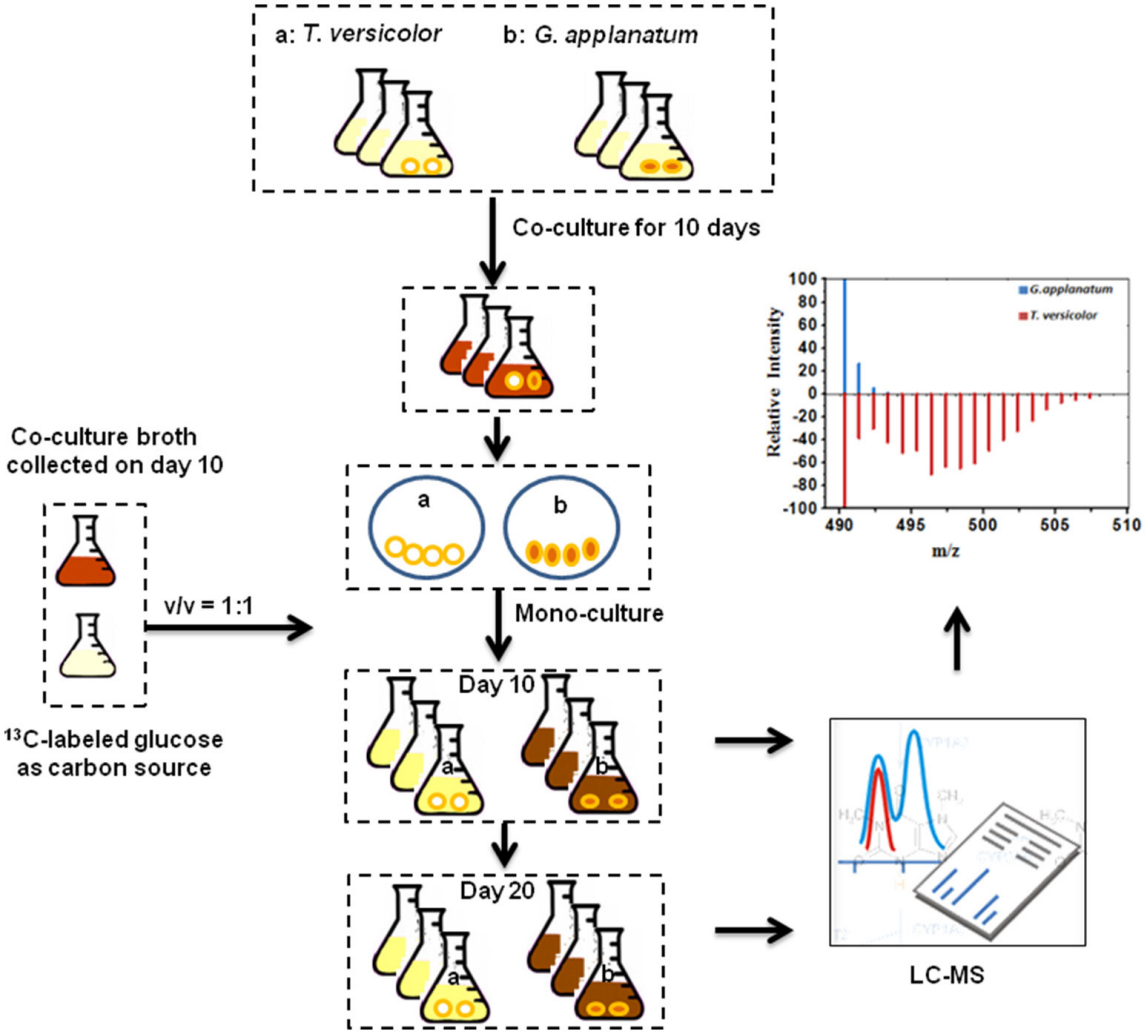
^13^C-labeling analysis procedure. *T. versicolor* and *G. applanatum* were co-cultivated for 10 days, and then their mycelia were sterilely separated and mono-cultivated in the liquid medium which contained half of fresh medium with ^13^C-labeled glucose as carbon source and half of co-cultured supernatants. Then samples were harvested in the mono-cultures of *T. versicolor* and *G. applanatum* and analyzed on LC-QTOF-MS.

To associate induced features with corresponding fungus, we initially harvested individual mycelium pellets of *T. versicolor* and *G. applanatum* after the co-culture for 10 days and then detected the abundance of *in vivo* induced features. Many of the induced features were observed for both samples of *T. versicolor* and *G. applanatum* (data not shown). This made it impossible to distinguish if individual or both fungi were induced to generate the compounds in the co-culture. To overcome this issue, we designed a ^13^C-labeling approach in the liquid co-cultivation. The workflow is shown in Figure 3. First, *T. versicolor* and *G. applanatum* were co-cultivated for 10 days to activate the cryptic genes. Next, their mycelia were sterilely separated and mono-cultivated in the liquid medium which contained half of fresh medium with ^13^C-labeled glucose as carbon source and half of co-cultured supernatants collected on day 10. Then, the samples were harvested in the mono-cultures of *T. versicolor* and *G. applanatum* on days 10 and 20 and analyzed by LC-QTOF-MS. In the preliminary experiments, the samples were also harvested after 5 days of mono-culture with the addition of the supernatants. However, many of induced features were only slightly labeled. It was likely due to a relatively long lag phase and low growth rate in the mono-culture of *T. versicolor* and *G. applanatum* after the stimulation of the co-culture. In addition, for the unlabeled features, ^13^C-labeling was not detected on day 5 either. Therefore, the samples were harvested later in order to obtain the strong MS signal. As the incorporation of ^13^C-labeled carbons from glucose increases the molecular weight of metabolites, the mass shift determined from the mass spectra provides then the information about which fungi generated the induced features.

**Figure 4:**
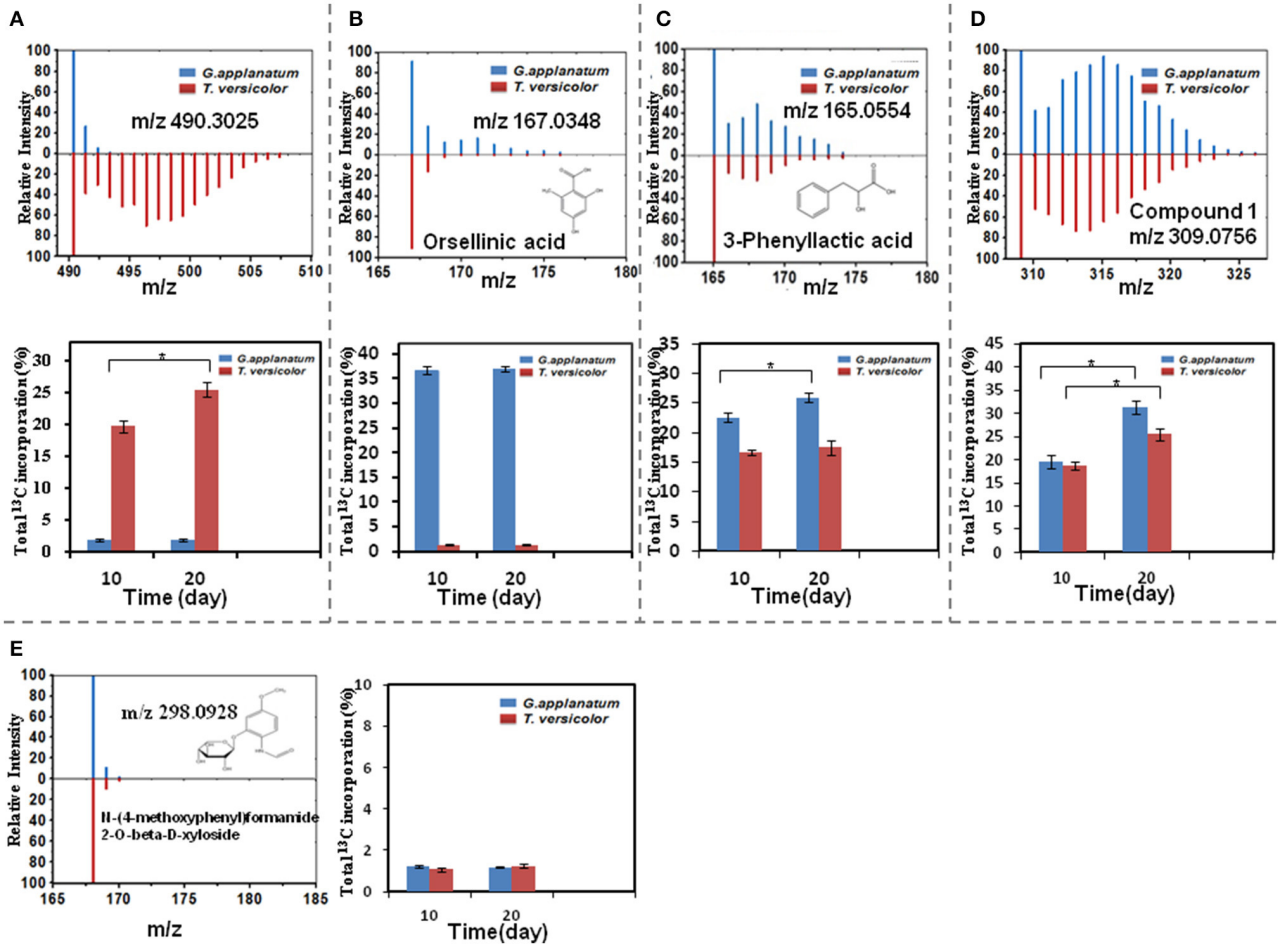
A comparison of relative MS intensity suggests that the induced features have ^13^C incorporation with time. **(A–D)** Upper figures: representative mass spectral data for 10 day time point showing that mass intensity caused by ^13^C incorporation in the mono-culture of *T. versicolor* or *G. applanatum*, Lower figures: comparison of total ^13^C incorporation shows that induced feature was produced by either *T. versicolor* or *G. applanatum*, or both. Data show the mean with error bars indicating standard deviation calculated from three independent replicates (^*^P ≤ 0.05, *t*-test). **(E)** There was no ^13^C incorporation for N-(4-methoxyphenyl)formamide 2-O-beta-D-xyloside on day 10 and 20.

Total 31 induced features were found to have ^13^C incorporation in the mono-culture, and 43 features did not incorporate any label (Figure 2). Among the labeled features activated by the co-culture, 20 originated from *T. versicolor* and 6 features were derived from *G. applanatum*. Five features were induced by both *T. versicolor* and *G. applanatum* (Figure 2). Several representative ^13^C labeling results are shown in Figure 4. In Figure 4A, the m/z 490 (experimental m/z 490.3025, predicted elemental component C_24_H_45_NO_9_, error 0.3 ppm) was a newly synthesized feature in the co-culture. As ^13^C-labeled carbon was incorporated into the feature m/z 490, intensity of m/z 491 (m/z + 1) up to m/z 506 (m/z + 16) increased. This change was observed only in the mono-culture of *T. versicolor* and indicates that m/z 490 was particularly induced in this species. The calculation of total ^13^C incorporation indicated that about 19 and 25% carbon was replaced with ^13^C on days 10 and 20, respectively (bottom part of Figure 4A). The m/z 167.0348 in Figure 4B was highly produced during the co-culture and identified as orsellinic acid in the previous publication (Yao et al., 2016). In contrast to the above example, the m/z 167 incorporated ^13^C-labels in the same time frame only in the mono-culture of *G. applanatum*, resulting in the parent ions shifted from m/z 167 to m/z 174 in the ^13^C-labeled mass spectra and total ^13^C incorporation level reached 14% on day 20 (Figure 4B). The m/z 165.0554 was previously identified as 3-phenyllactic acid (Yao et al., 2016), and ^13^C-labeled mass spectra ranging from m/z 165 to m/z 174 were observed in both mono-cultures of *T. versicolor* and *G. applanatum* (Figure 4C). Similar example, feature with m/z 309 (experimental m/z 309.0756, C_18_H_14_O_5_, error 0.37 ppm) increased 15.4-fold during the co-culture in comparison with MS signal in the control mono-culture and ^13^C-labeling was observed for both fungi (Figure 4D). It is worth mentioning that a contamination from the undesired fungus cannot be absolutely excluded during the transfer from the co-culture to the mono-culture, but that it does not affect the identification of the fungus producing a feature only found in the mono-culture of either *T. versicolor* or *G. applanatum* based on the ^13^C-labeling analysis.

**Figure 5:**
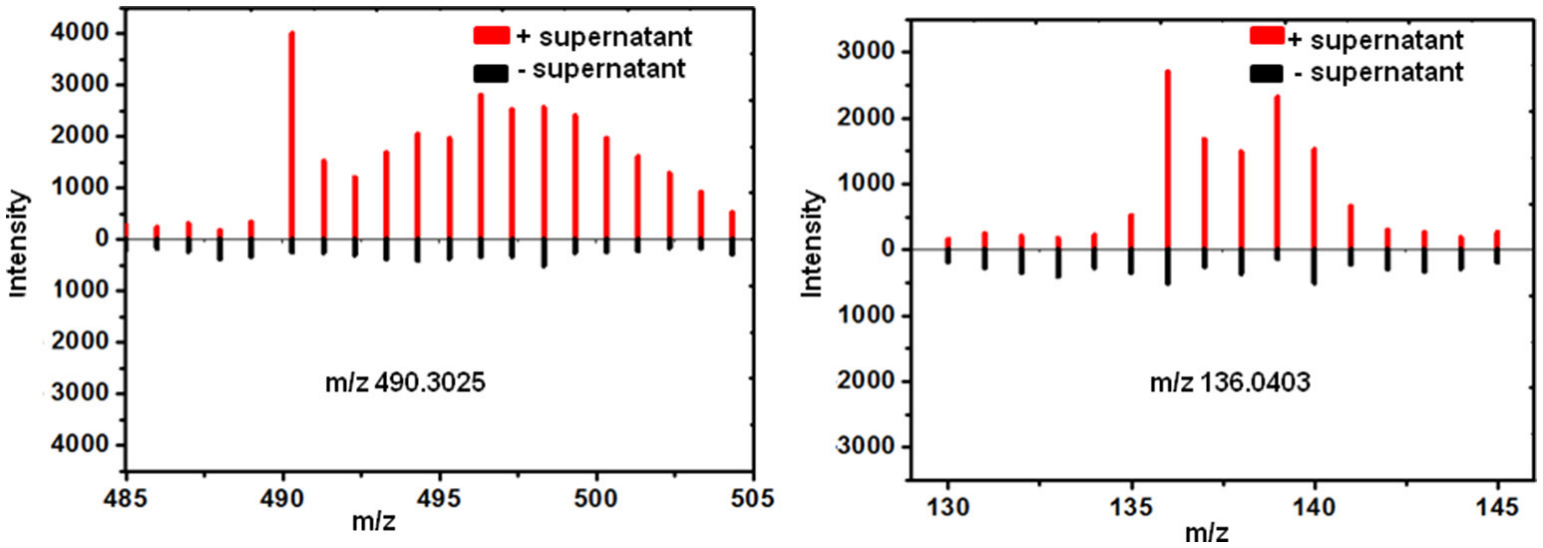
^13^C incorporation was not observed for the induced features on day 20 in the mono-culture of *T. versicolor* **(Left)** or *G. applanatum* **(Right)** without the addition of the supernatant of co-culture.

^13^C incorporation could be due to the induction of features which have been released into the medium during the co-culture. To confirm whether diffusible features were involved in triggering the silent gene expression in this study, we treated *T. versicolor* and *G. applanatum* with fresh medium containing ^13^C labeled glucose as carbon source but without the addition of the supernatant of co-culture on day 10. As shown in Figure 5 as an example, ^13^C labeling of m/z 490.3025 and 136.0403 was not observed up to 20 days but when the supernatant was added the features were significantly labeled. Notably, for 12 induced features with high signal intensities, including newly synthesized xyloside [N-(4-methoxyphenyl)formamide 2-O-beta-D-xyloside; Yao et al., 2016], we did not detected any ^13^C incorporation in the mono-culture of either fungus (Table 1, Figure 4E, Supplementary Figure 1D). Therefore, it was not possible to assign their origin to a specific fungus.

### Dereplication of newly discovered features by molecular network analysis

**Figure 6:**
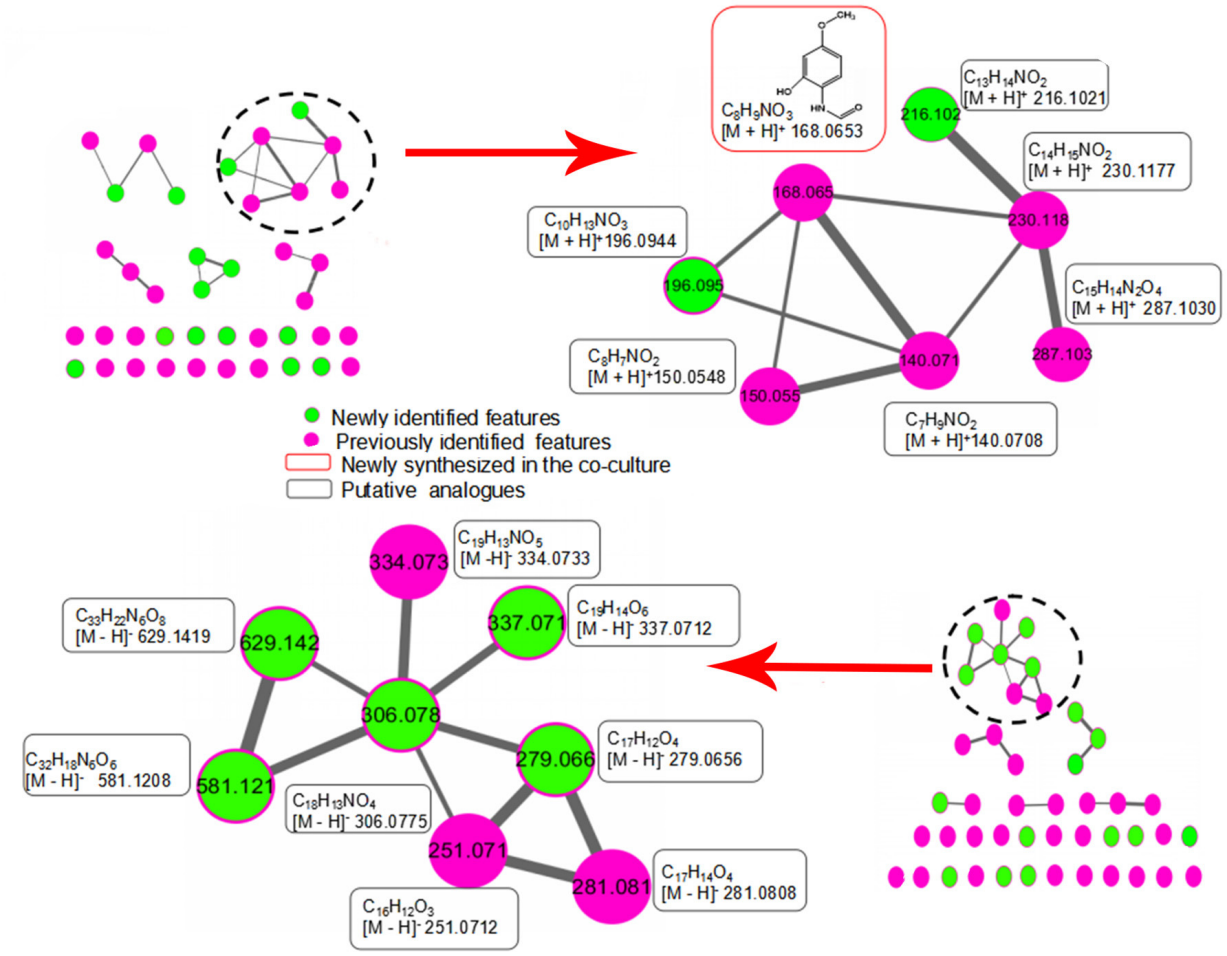
Molecular network analysis of newly discovered features in the co-culture. The green nodes represent parent ions of newly identified features and purple nodes are the previous features, and the thickness of the edges between nodes indicates the degree of similarity between their respective MS/MS spectra. Upper part shows a constructed subnetwork to dereplicate m/z 196.0944 and 216.1021. Lower part shows five newly identified features (m/z 279.0656, 306.0775, 337.0712, 581.1208, 629.1419) and three previous features (m/z 251.0712, 281.0808 and 334.0733) that clustered together.

To investigate the structural similarities occurring for 28 newly discovered features in this study, MS/MS fragmentation spectra of the induced features in Table 1 were processed and organized as molecular network with the previously identified features. Figure 6 (upper part) shows a constructed subnetwork to dereplicate m/z 196.0944 and 216.1021. These two new features likely possessed similar backbone structure with the previously identified N-(2-hydroxy-4-methoxyphenyl) formamide (m/z 168.0653) and its analogous features (m/z 140.0708, 150.0548, 230.1177, 287.1030) due to their close fragmentation patterns (Supplementary Figure 2). This data was also in agreement with ^13^C-labeling analysis, in which most of features involved in this subnetwork were not labeled either. In another example, five newly identified features and three previous features (m/z 251.0712, 281.0808, and 334.0733) were clustered together with high scores (Figure 6, bottom part). Comparison of the fragment ions of m/z 306.0775, 334.0733, 581.1208, 629.1419, and 279.0656 showed some common ions of m/z 77.04, 117.03, 235.08 which were likely derived from fragmentation of m/z 279.0656, suggesting that these features probably had the same backbone structure and belonged to a series of structural derivatives (Supplementary Figure 2). Moreover, ^13^C incorporation of m/z 279.0656, 251.0712, and 281.0808 were clearly detected in the mono-culture of *T. versicolor* with the addition of the supernatant of co-culture (Supplementary Figure 1), suggesting that the derivatives of m/z 279.0656 (i.e., m/z 306.0775, 334.0733, 581.1208, and 629.1419, which did not show ^13^C incorporation due to the weak signals in the mono-culture) were also likely biosynthesized by *T. versicolor* under the co-cultured condition.

### Identification of induced compound 1 and analysis of its biological activity

**Figure 7:**
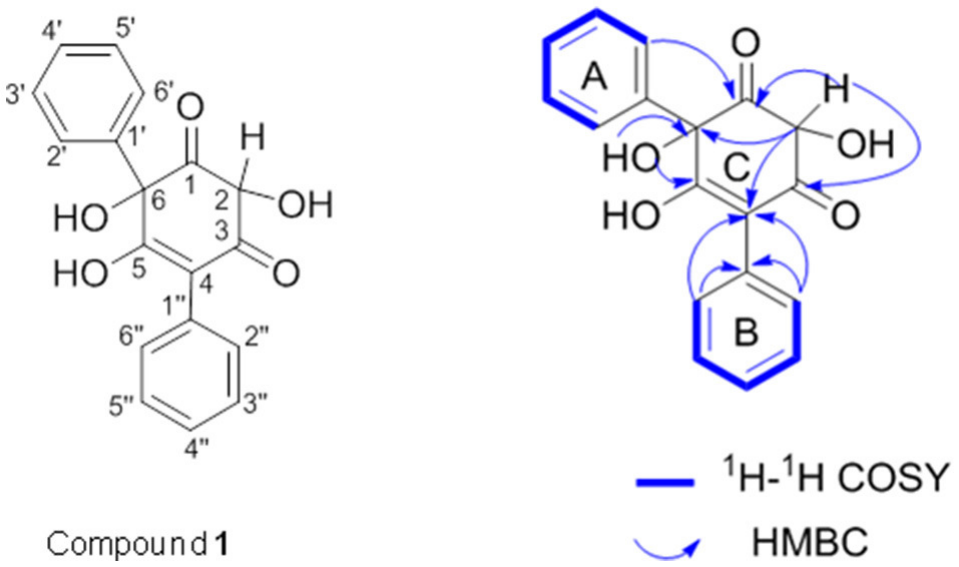
1H−1H COSY (blue bold lines) and key HMBC (blue arrows) correlations of compound **1**. COSY is two-dimensional spectrum which is used to identify spins coupled to each other, and HMBC is two-dimensional inverse H,C correlation technique that allow for the determination of carbon to hydrogen connectivity.

Since compound **1** was derived from both *T. versicolor* and *G. applanatum* (Figure 4D), and 15.4-fold more abundant in the co-culture, we isolated and purified sufficient amount for detailed characterization. Compound **1** was a pale yellow powder with the molecular formula of C_18_H_14_O_5_. The ^1^H, ^13^C-NMR and HSQC spectrum showed the presence of two carbonyl carbon, one oxygen connected CH, one oxygen connected to quaternary carbon, two single benzene rings and two olefinic carbon atoms (one with oxygen attached to it) (Supplementary Table 1 and Supplementary Figure 3). All the information suggested that the basic skeleton of the compound **1** is a terphenyl derivative with two mono-substituted benzene rings. The COSY spectrum showed the ^1^H–^1^H spin systems of H-2′/H-3′/H-4′/H-5′/H-6′ and H-2″/H-3″/H-4″/H-5″/H-6″, assigned two mono-substituted benzene rings (A and B). The HMBC correlations from H-2 (δ_H_ 4.49) to C1 (δ_C_ 203.4), C3 (δ_C_ 197.4), C4 (δ_C_ 113.5), and C6 (δ_C_ 90.9), and from OH-6 (δ_H_ 5.44, dimethylsulfoxide (DMSO-*d*_6_) to C1 (δ_C_ 202.45), C5 (δ_C_ 191.28) and C6 (δ_C_ 90.9) established the ring C. The fragment ring A was linked to C-6 of ring C supported by the HMBC correlations from H-6' (δ_H_ 7.98) to the carbonyl C1 (δ_C_ 203.4). Likewise, the linkage of the ring B to the ring C at C-4 was confirmed by HMBC correlations from H-2″ (δ_H_ 7.88) to C4 (δ_C_ 113.5) and C-1″(δ_C_ 135.4). Therefore, the structure of compound **1** was identified as a phenyl polyketide, 2,5,6-trihydroxy-4,6-diphenylcyclohex-4 -ene-1,3-dione (Figure 7).

**Figure 8:**
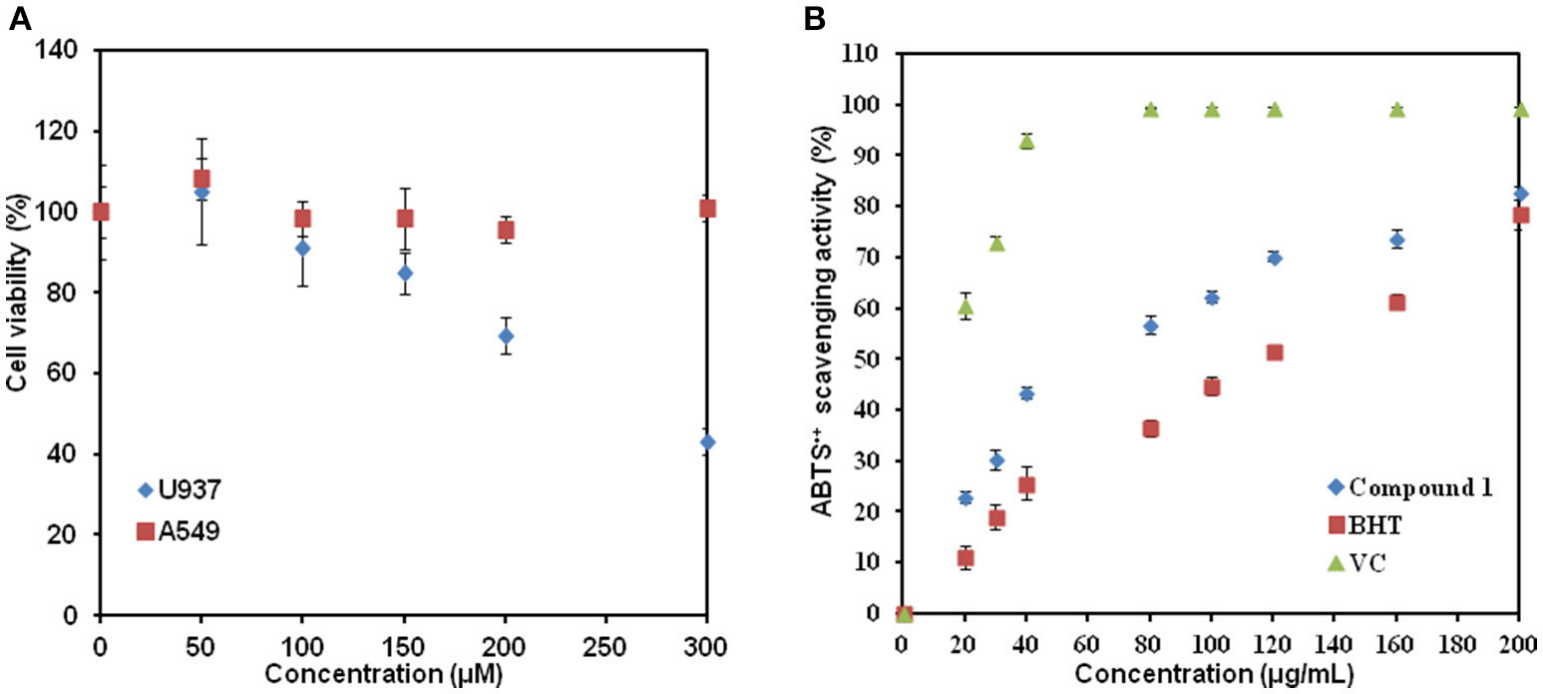
The biological activity study of compound **1**. **(A)** Addition of compound **1** affected the cell viability of U937 but not of A549. **(B)** Graph of ABTS^•+^ scavenging activity vs. concentration. Data show the mean with error bars indicating standard deviation calculated from three independent biological replicates. VC, ascorbic acid; BHT, 2,6-di-*tert*-butyl-4-methoxyphenol.

We further tested biological activity of compound **1**. The human lung cancer cell lines A549 and leukemic cell lines U937 were treated with compound **1** at various concentrations for 48 h. As shown in Figure 8A, no visible changes in cell viability were detected for human lung cancer cell line A549 when the concentrations were increased to 300 μM. In contrast, compound **1** inhibited the viability of leukemic cells in a dose-dependent manner. The IC_50_ at 48 h was determined to be 276 ± 5 μM (equal to 85 mg/L). In addition, based on the structural characteristics of compound **1**, we also studied whether compound **1** had antioxidant properties using ABTS assay (Figure 8B). The highest percentage of antioxidant capacities (82.65 ± 1.25%) was observed for compound **1** at the concentration of 200 μg/mL. This is comparable with the report of crude extracts from berries (Abu-Bakar et al., 2016). The comparison of the antioxidant activity with ascorbic acid (VC) and phenolic antioxidant 2,6-di-*tert*-butyl-4-methoxyphenol (BHT) revealed that ascorbic acid> compound **1** > BHT.

## Discussion

Determining the origin of induced features in the co-culture remains a large challenge, because it generally requires the chemical structures with their biological information. Previously Bertrand et al. discovered five *de novo* induced compounds from the co-culture of *Trichophyton rubrum* and *Bionectria ochroleuca*, and elucidated the origin of one of them based on its non-sulfated form detected in the mono-culture of *B. ochroleuca* (Bertrand et al., 2013). However, other four compounds could not be associated with corresponding fungi due to the lack of their structures. In another study Ola et al. isolated nine compounds and speculated that four of them detected only in the co-culture originated from *Fusarium tricinctum* in terms of structural analogies with the known fungal products from the Xylariaceae family, but the producer of remaining five compounds was still unclear because of insufficient biochemical evidences (Ola et al., 2013). In this work, among 74 induced features, 31 features were shown to be produced specifically by either *T. versicolor* or *G. applanatum*, or by both fungi using a ^13^C-based labeling analysis. This methodology was able to distinguish the origin even if the identities of compounds were not available or almost nothing was known about biochemical aspects. In more detail, the total ^13^C incorporation in the same fungal culture varied noticeably among features. Isotopic steady state is the state that ^13^C-labeling signatures in metabolites become time invariant (Antoniewicz et al., 2007). In current work, ^13^C incorporation into 20 features (e.g., orsellinic acid) had the similar abundance levels between days 10 and 20 and these features had the similar ^13^C-labeling pattern (data not shown), indicating that they likely reached isotopic steady state around 10 days (Figure 4B, Supplementary Figure 1). In contrast, ^13^C incorporation level of 11 features including novel phenyl polyketide (compound **1**) increased from day 10 to day 20 (Figures 4A,C,D, Supplementary Figure 1), suggesting either the fluxes via their synthetic pathways were low or their metabolite pools were relatively large (Zamboni et al., 2009).

By comparison of ^13^C-labeling patterns between with and without the addition of the supernatant of co-culture, it was demonstrated that the induction of gene expression and synthesis of corresponding metabolites during the co-culture of *T. versicolor* and *G. applanatum* depended indeed on the signaling molecules released into the medium. Forty-three induced features in the supernatant of co-culture on day 10 were detected with signal intensity ranging from 10^3^ to 10^5^ (Table 1), but in the current work we were not able to determine which features were responsible for signaling to activate gene expression in *T. versicolor* or *G. applanatum*. Additional studies will be needed to confirm this link as well as to elucidate the structure and function of signaling molecules. Notably, the previous report also demonstrated that an intimate physical interaction of the actinomycete *S. hygroscopicus* and fungal mycelia of *Aspergillus nidulans* was required to induce specific stimulation of the silent polyketide synthases and non-ribosomal peptide synthetases gene clusters (Schroeckh et al., 2009). In our research, we found 12 highly produced features that did not incorporate ^13^C labeling in the mono-culture. One potential explanation is that these features were also produced in the co-culture via mycelium physical interaction to elicit the specific response. In addition, compound **1** had over 15-fold higher MS signal intensity in the co-culture than in the control mono-culture of *T. versicolor* where the mycelia were not pre-induced by the co-culture. It was not detected in the control mono-culture of *G. applanatum*, yet ^13^C incorporation in *G. applanatum* was 1.3-fold higher than that in *T. versicolor* on day 20 (Figure 4D). This finding suggests that the co-culture could significantly activate the encoding genes or gene clusters related to the synthesis of Compound **1** in *G. applanatum*. This provides an important insight into possible manipulation of *G. applanatum* to enhance biosynthesis of novel metabolites.

Comparing MS fragmentation similarity including common losses, molecular network analysis is able to obtain a simultaneous visual investigation of identical molecules, analogs, or metabolite families, thereby assisting the structural analysis (Winnikoff et al., 2014; Cabral et al., 2016). In our previous research, this method was utilized to show that the common neutral loss of 132 Dalton resulted from the deglycosylation reaction, which helped to find a series of novel xylosides generated during the co-culture (Yao et al., 2016). Here, combined molecular network analysis and ^13^C-labeling analysis suggested further that some newly discovered features were not only structurally analogous but also had similar induction mechanism and were likely produced by the same fungus. Thus, combination of network analysis and ^13^C labeling shows promise to accelerate the elucidation of biosynthetic pathways of novel metabolites.

Several type II polyketides have been reported to be high-value medicals (Zhang W. et al., 2012). In antioxidant assay, compound **1** had better activity than BHT. More interestingly, compound **1** at micromolar concentration was able to inhibit the viability of leukemic cells. For comparison, Zhang et al. reported that matrine extracted from *Sophora flavescens* inhibited the proliferation of acute myeloid leukemia cell lines U937 in a dose- and time-dependent manner with the IC_50_ of 590 mg/L at 24 h, and resulted in the maximal apoptosis rates with 37.2% for 24 h (Zhang S. et al., 2012). Wang et al. also demonstrated that 100 μg/mL of *Ganoderma lucidum* extracts could greatly suppress leukemic cell growth with the inhibition rate of 75% (Wang et al., 1997). In our case, the IC_50_ of compound **1** was at the same level with matrine and crude extracts from *G. lucidum*. Thus, it can become a starting point for development of lead compounds to cure leukemia or other cancers.

## Conclusion

The application of ^13^C-labeling analysis produced valuable insight into the role of individual partners in the co-culture in production of known or unknown induced metabolites. Moreover, ^13^C-labeling approach combined with molecular network analysis can reveal whether certain structural analogs were produced by the same fungus or through the similar activation mechanism. The current ^13^C-labeling information sets an important foundation for further studies in the basidiomycetes, including but not limited to novel metabolites discovery and biosynthetic capacity improvement.

## Author contributions

X-YX, BQ, and SY: conceived and designed the project; X-YX, X-TS, X-JY, Y-MZ, HF, and JY: performed the experiments; X-YX, X-TS, L-PZ, F-YD, MS, and SY: interpreted the data. All authors contributed to the preparation of the manuscript, read and approved the final manuscript.

### Conflict of interest statement

The authors declare that the research was conducted in the absence of any commercial or financial relationships that could be construed as a potential conflict of interest. The reviewer MT and handling Editor declared their shared affiliation.
