# Supplementary material for: Metabolomics Investigation of an Association of Induced Features and Corresponding Fungus during the Co-culture of Trametes versicolor and Ganoderma applanatum
Source: Front Microbiol. 2018 Jan 9;8:2647. doi: 10.3389/fmicb.2017.02647 (PMC5767234; doi:10.3389/fmicb.2017.02647)
Supplement: Supplementary file 1 [file Table1.PDF]

Supplementary Table 1. ^{1}H-NMR and ^{13}C-NMR data for compound **1**, δ in ppm

Position	Compound 1			
	δ_C (CD_3OD)	δ_H (CD_3OD)	δ_H (DMSO-d_6)	δ_C (DMSO-d_6)
1	203.4			202.45
2	82.2	4.49 (s)	4.13(s)	80.97
3	197.4			194.4
4	113.5			104.83
5	193.9			191.28
6	90.9			89.34
1'	138.9			138.06
2',6'	130.8	7.98 (d, J=7.8Hz)	7.93 (d, J=7.5Hz)	129.69
3',5'	128.6	7.35 (t, J=7.8Hz)	7.36 (t, J=7.8Hz)	127.21
4'	132.9	7.45 (m)	7.45 (t)	131.19
1''	135.4			137.13
2'',6''	128.4	7.88 (d, J=7.5Hz)	8.44 (d, J=7.8Hz)	124.28
3'',5''	128.5	7.26 (t, J=7.5Hz)	7.14 (t, J=7.5Hz)	126.91
4''	125.9	7.08 (m)	6.89 (t)	122.27
2-OH			4.69 (s)	
5-OH			16.2 (brs)	
6-OH			5.44 (s)	
