# Supplementary material for: Metabolomics Investigation of an Association of Induced Features and Corresponding Fungus during the Co-culture of Trametes versicolor and Ganoderma applanatum
Source: Front Microbiol. 2018 Jan 9;8:2647. doi: 10.3389/fmicb.2017.02647 (PMC5767234; doi:10.3389/fmicb.2017.02647)
Supplement: Supplementary file 2 [file Image1.PDF]

Supplementary Figure 1. A comparison of total ^{13}C incorporation of induced features between the mono-culture of *T. versicolor* and *G. applanatum* on days 10 and 20 after the stimulation by their co-culture. (A) Induced features were labeled in the mono-culture of *T. versicolor*; (B) Induced features were labeled in the mono-culture of *G. applanatum*; (C) Induced features were labeled in the mono-cultures of both *T. versicolor* and *G. applanatum*; (D) Induced features were not labeled. Data show the mean with error bars indicating standard deviation calculated from at least three independent biological replicates (*, $P \leq 0.05$; t-test).

(A)

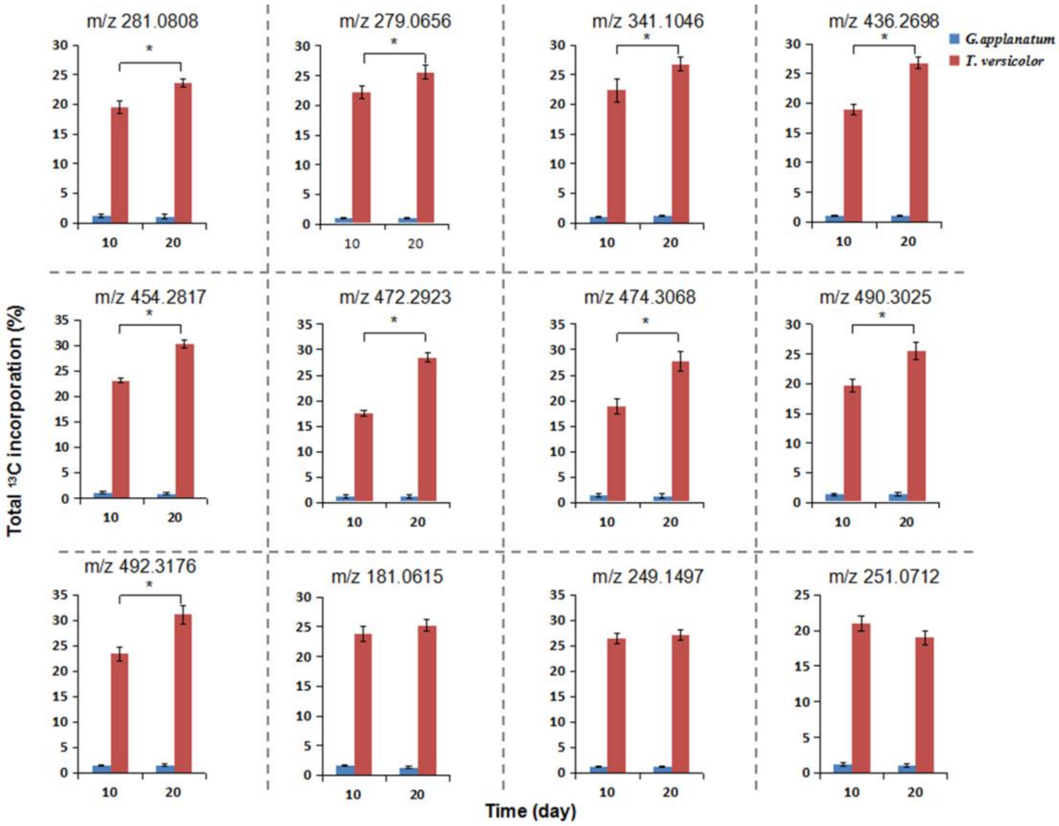

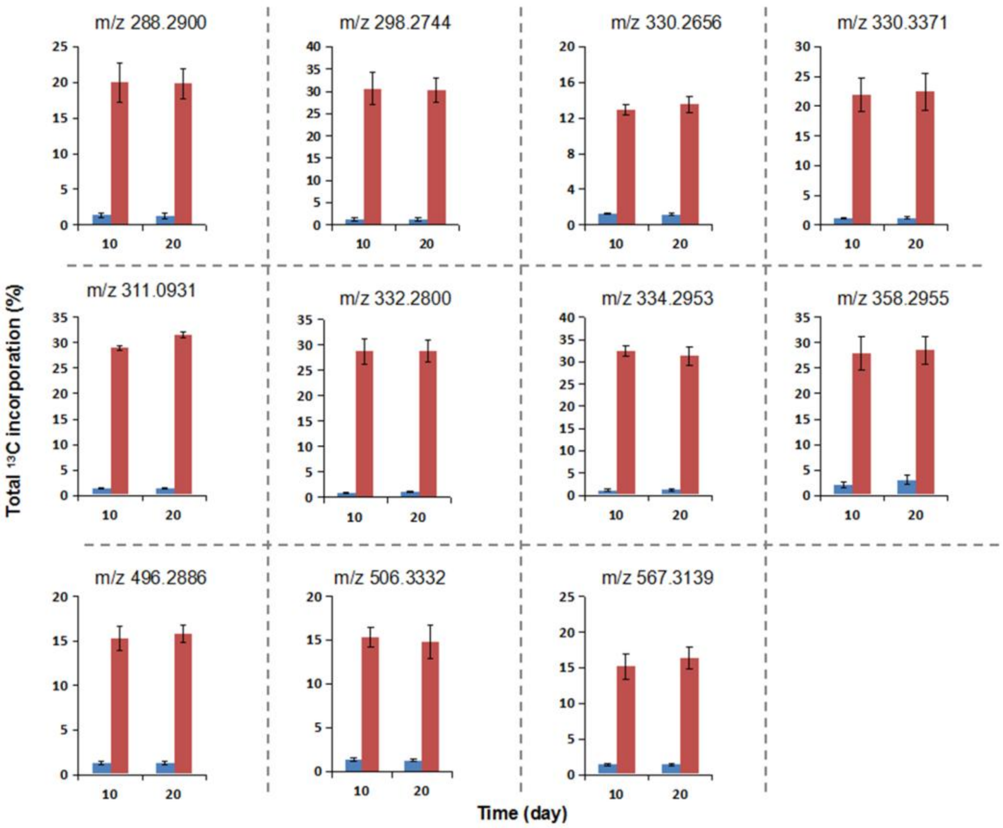
m/z 288.2900
m/z 298.2744
m/z 330.2656
m/z 330.3371
m/z 311.0931
m/z 332.2800
m/z 334.2953
m/z 358.2955
m/z 496.2886
m/z 506.3332
m/z 567.3139
Total ¹³C incorporation (%)
Time (day)

(B)

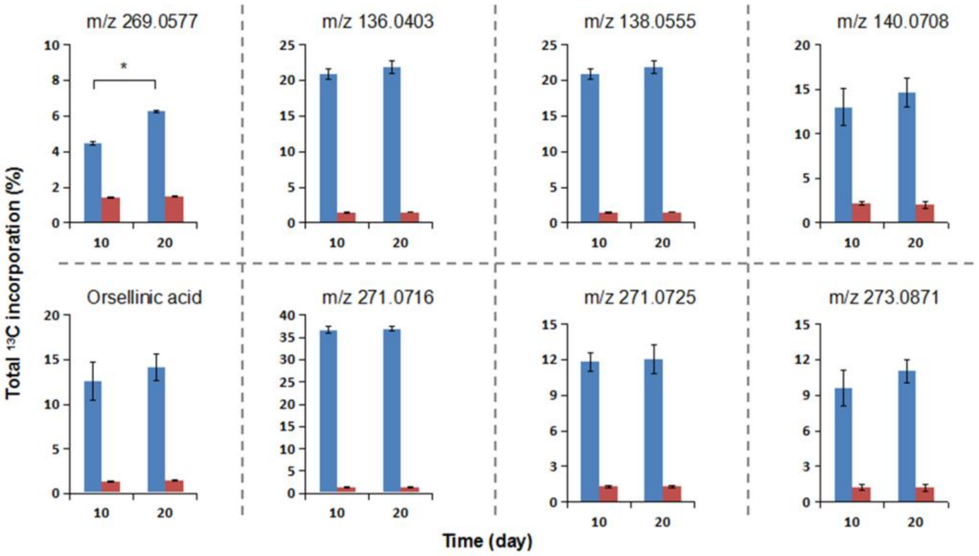
m/z 269.0577
m/z 136.0403
m/z 138.0555
m/z 140.0708
Orsellinic acid
m/z 271.0716
m/z 271.0725
m/z 273.0871
Total ¹³C incorporation (%)
Time (day)

(C)

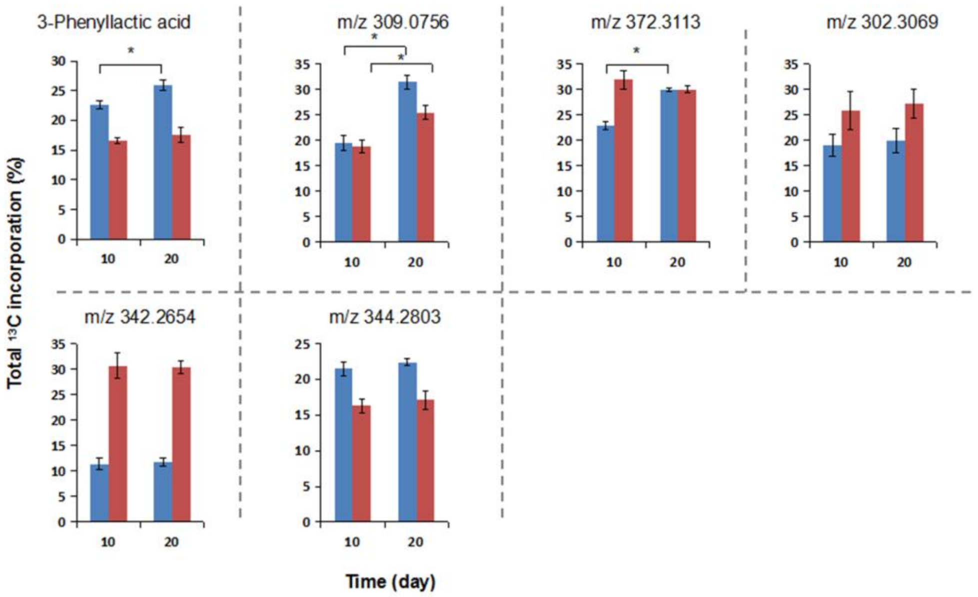

3-Phenyllactic acid
m/z 309.0756
m/z 372.3113
m/z 302.3069
m/z 342.2654
m/z 344.2803
Total ^{13}C incorporation (%)
Time (day)

(D)

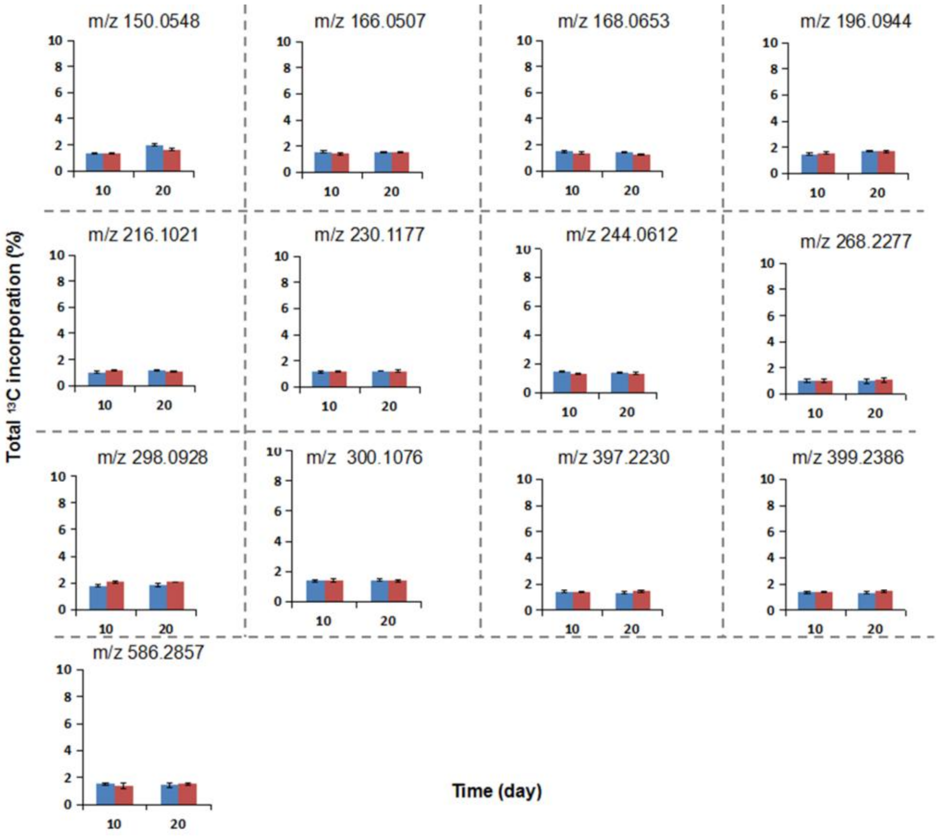

m/z 150.0548
m/z 166.0507
m/z 168.0653
m/z 196.0944
m/z 216.1021
m/z 230.1177
m/z 244.0612
m/z 268.2277
m/z 298.0928
m/z 300.1076
m/z 397.2230
m/z 399.2386
m/z 586.2857
Total ^{13}C incorporation (%)
Time (day)
