# Supplementary material for: Metabolomics Investigation of an Association of Induced Features and Corresponding Fungus during the Co-culture of Trametes versicolor and Ganoderma applanatum
Source: Front Microbiol. 2018 Jan 9;8:2647. doi: 10.3389/fmicb.2017.02647 (PMC5767234; doi:10.3389/fmicb.2017.02647)

Supplementary Figure 2.The MS/MS spectra of induced features involved in molecular network analysis.

MS/MS of m/z(+) 140.0708

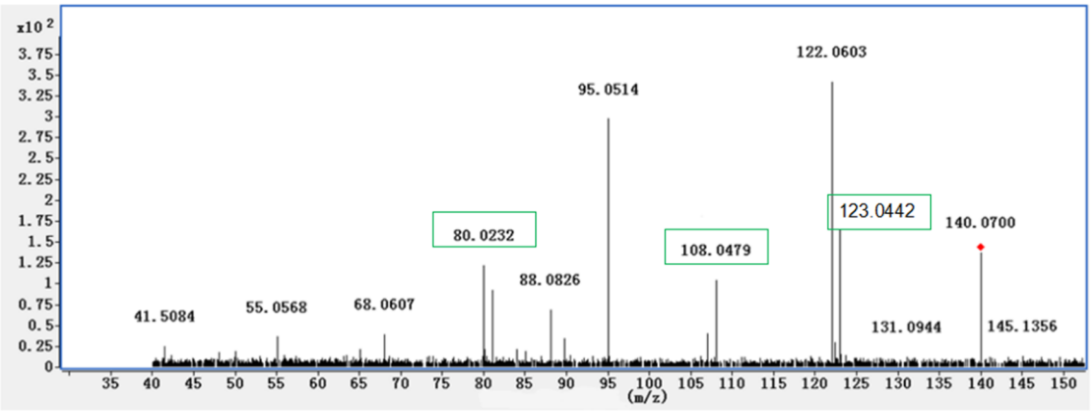

MS/MS of m/z(+) 150.0548

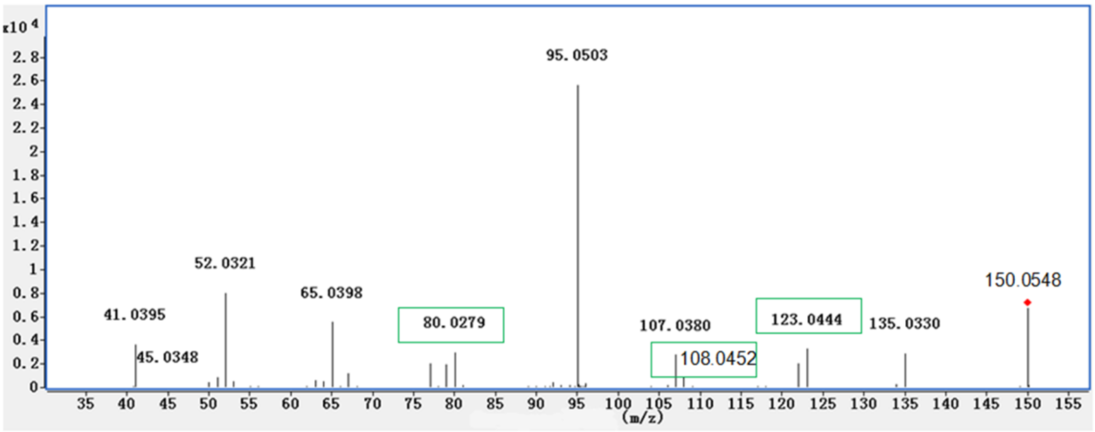

MS/MS of m/z(+) 168.0653

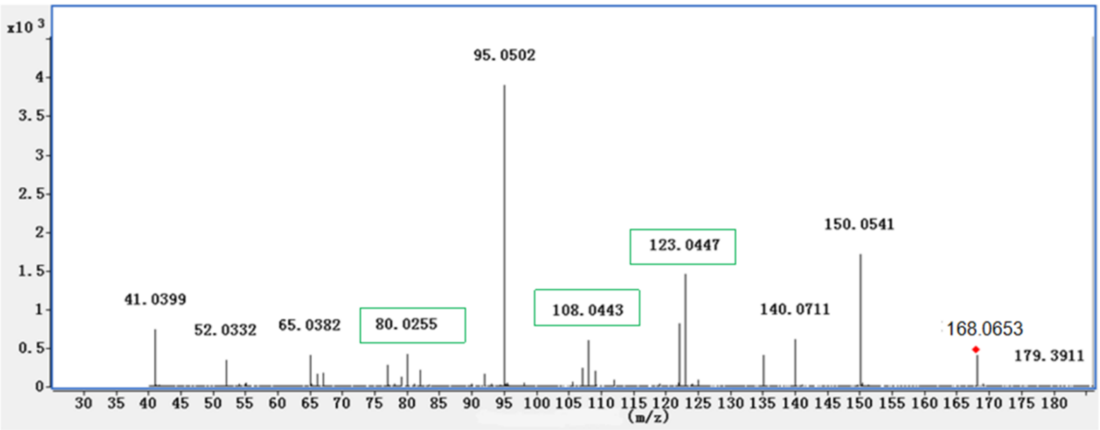

MS/MS of m/z(+) 196.0944

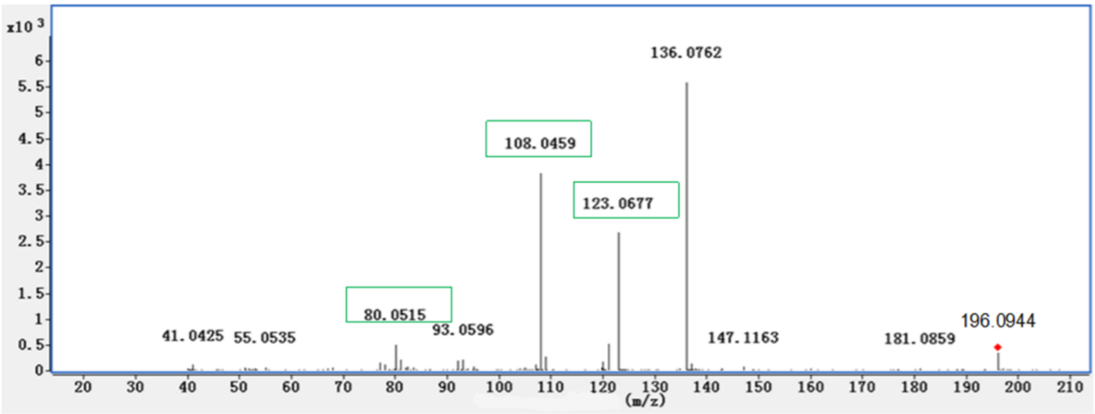

MS/MS of m/z(+) 216.1021

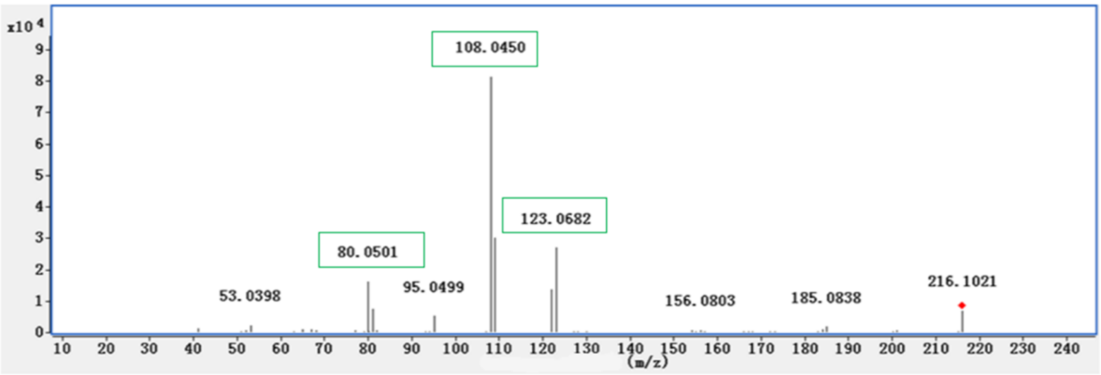

MS/MS of m/z(+) 230.1177

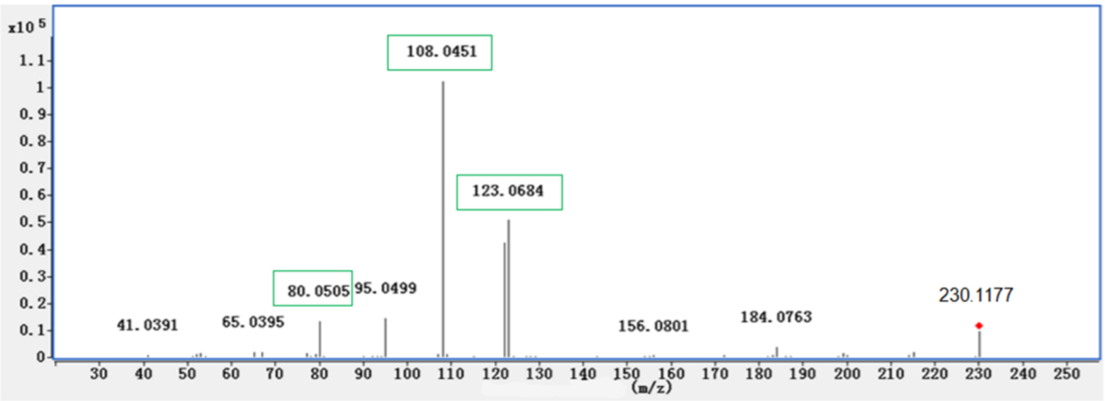

MS/MS of m/z(+) 287.1030

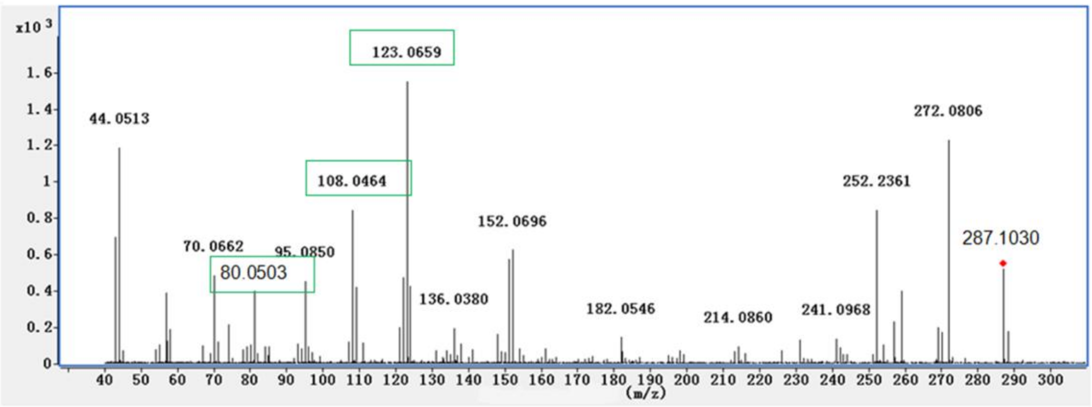

MS/MS of m/z(-) 251.0712

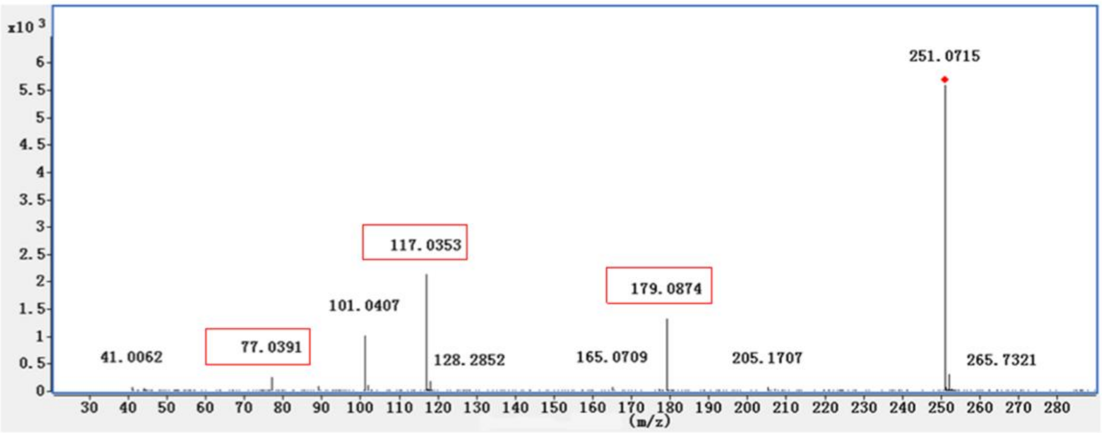

MS/MS of m/z(-) 279.0656

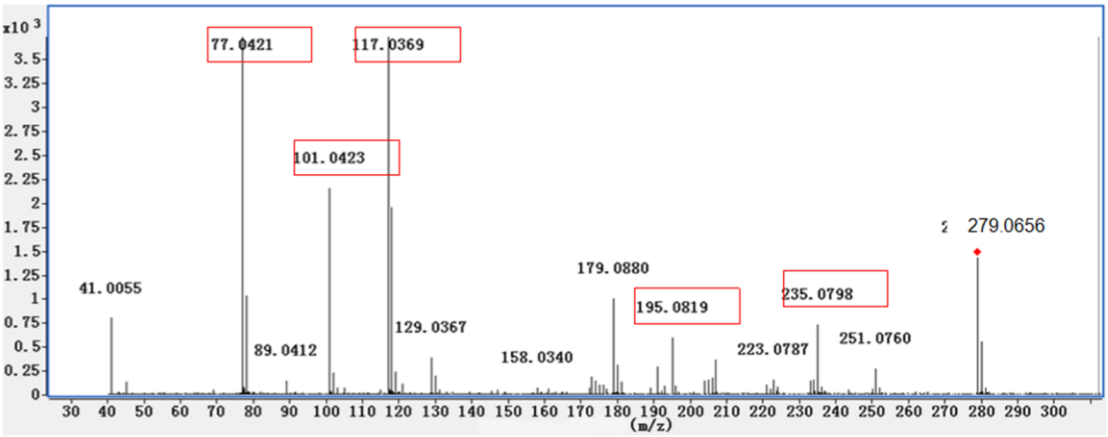

MS/MS of m/z(-) 281.0808

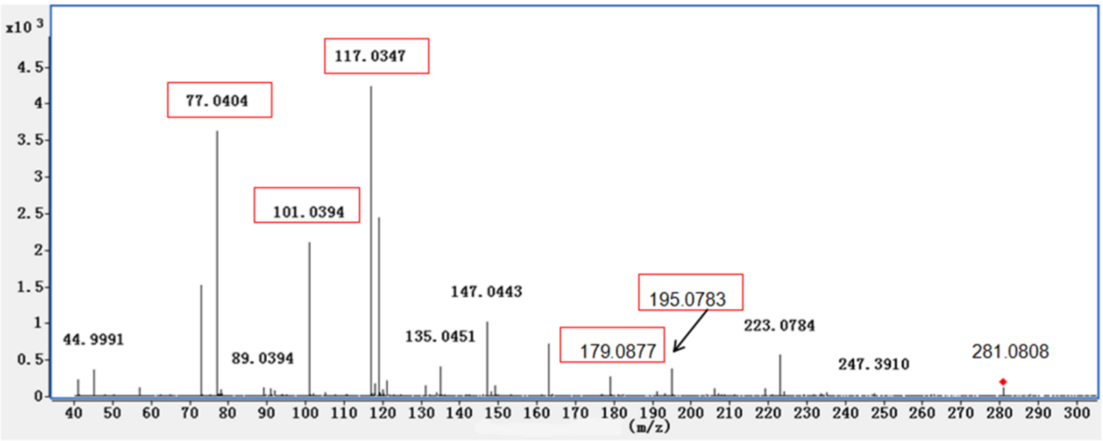

MS/MS of m/z(-) 306.0775

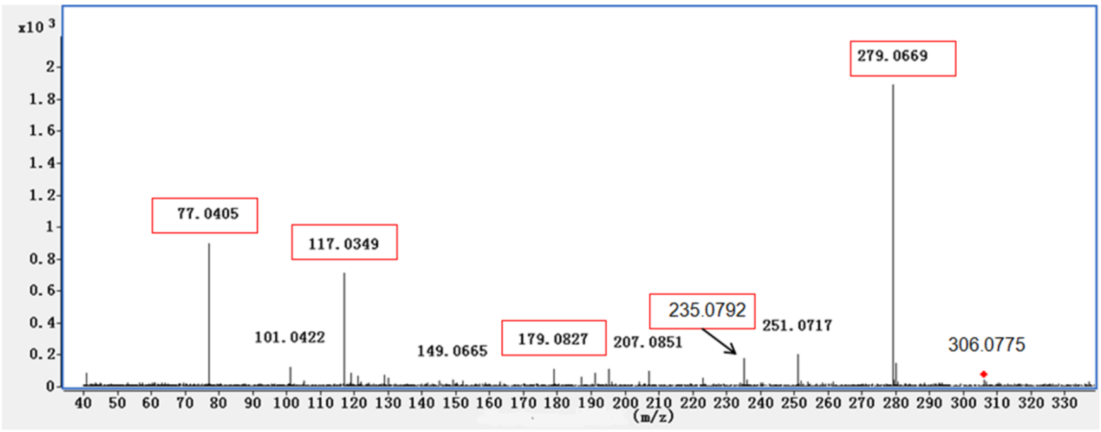

MS/MS of m/z(-) 334.0733

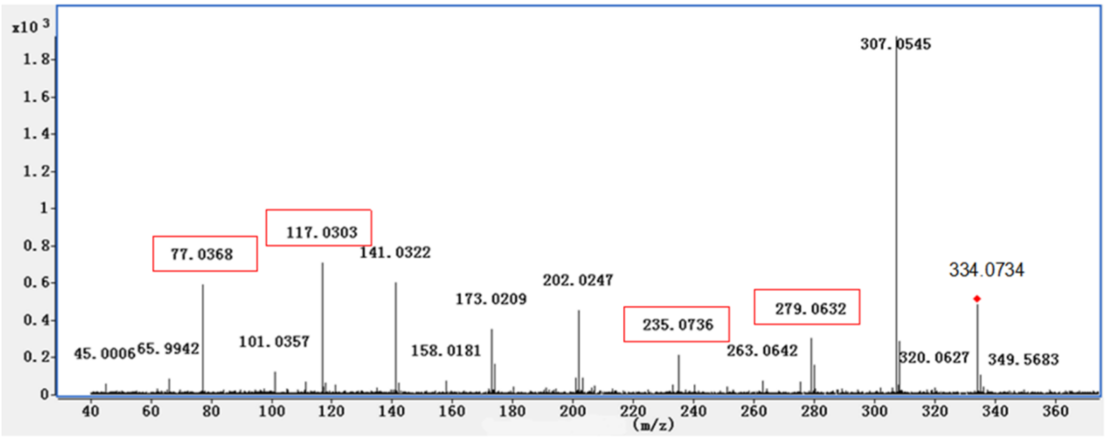

MS/MS of m/z(-) 337.0712

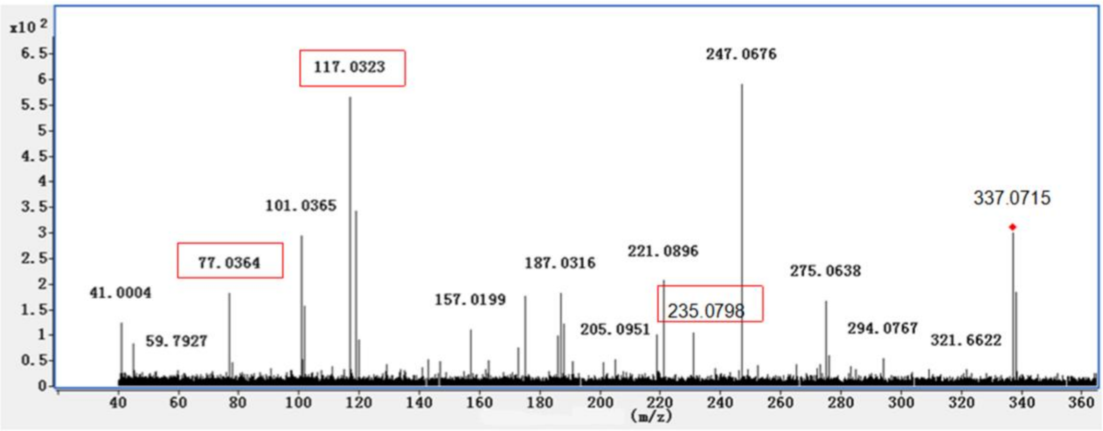

MS/MS of m/z(-) 581.1208

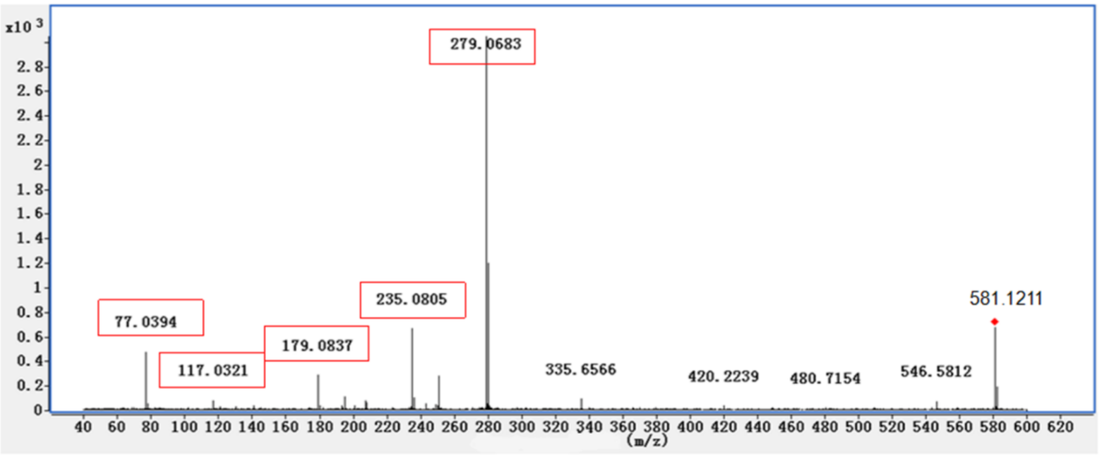

MS/MS of m/z(-) 629.1419

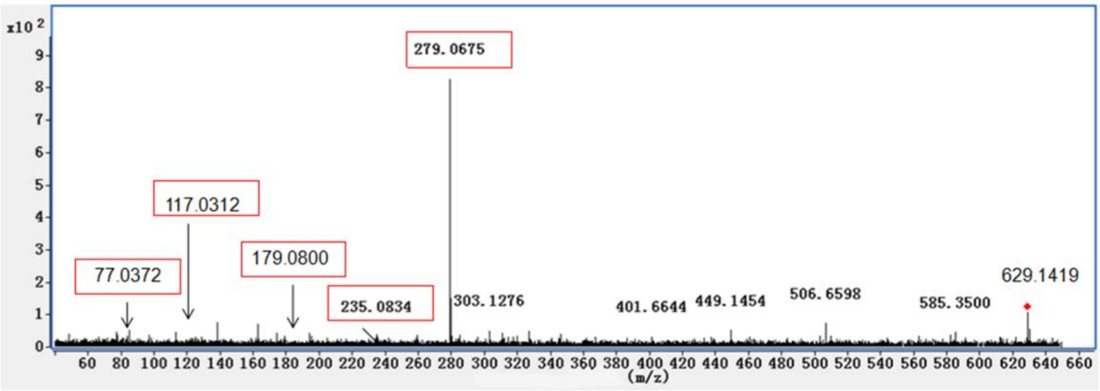

Supplement: Supplementary file 3 [file Image2.PDF]
