# Supplementary material for: Metabolomics Investigation of an Association of Induced Features and Corresponding Fungus during the Co-culture of Trametes versicolor and Ganoderma applanatum
Source: Front Microbiol. 2018 Jan 9;8:2647. doi: 10.3389/fmicb.2017.02647 (PMC5767234; doi:10.3389/fmicb.2017.02647)
Supplement: Supplementary file 4 [file Image3.PDF]

Supplementary Figure 3. NMR spectra of compound **1**

NMR spectrum of compound 1 (methanol)

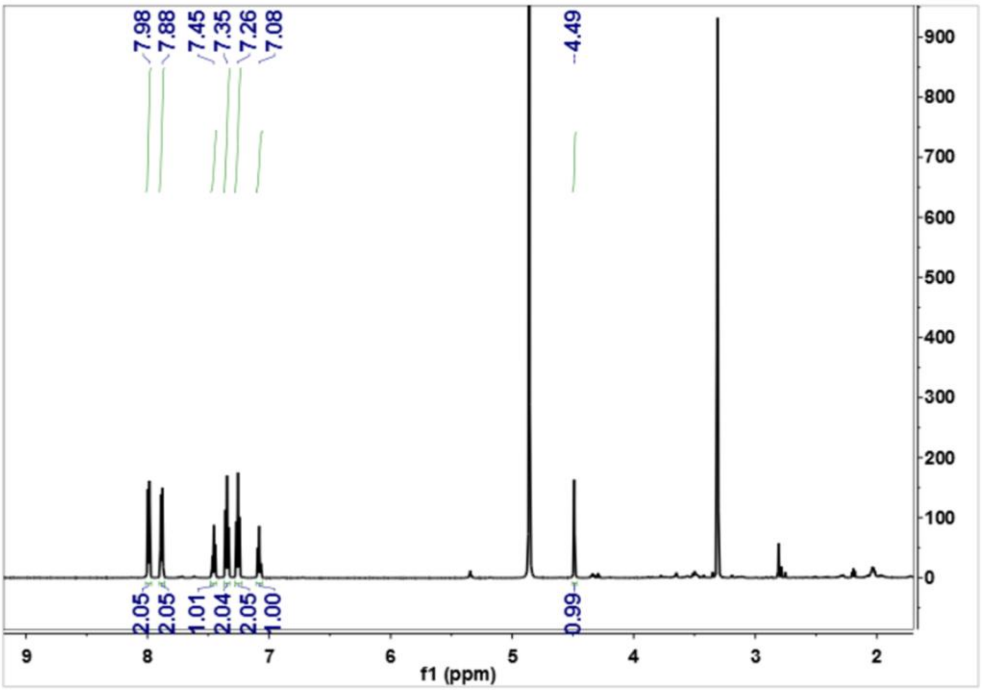

^{1}H-spectrum of compound 1

NMR spectrum of compound 1 (methanol)

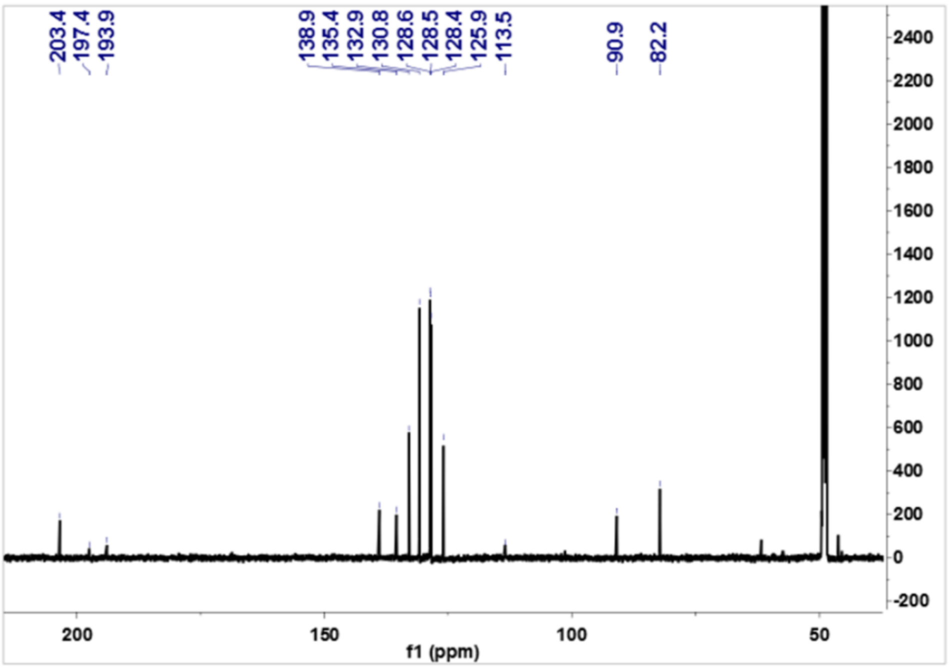

^{13}C-spectrum of compound 1

NMR spectrum of compound 1 (methanol)

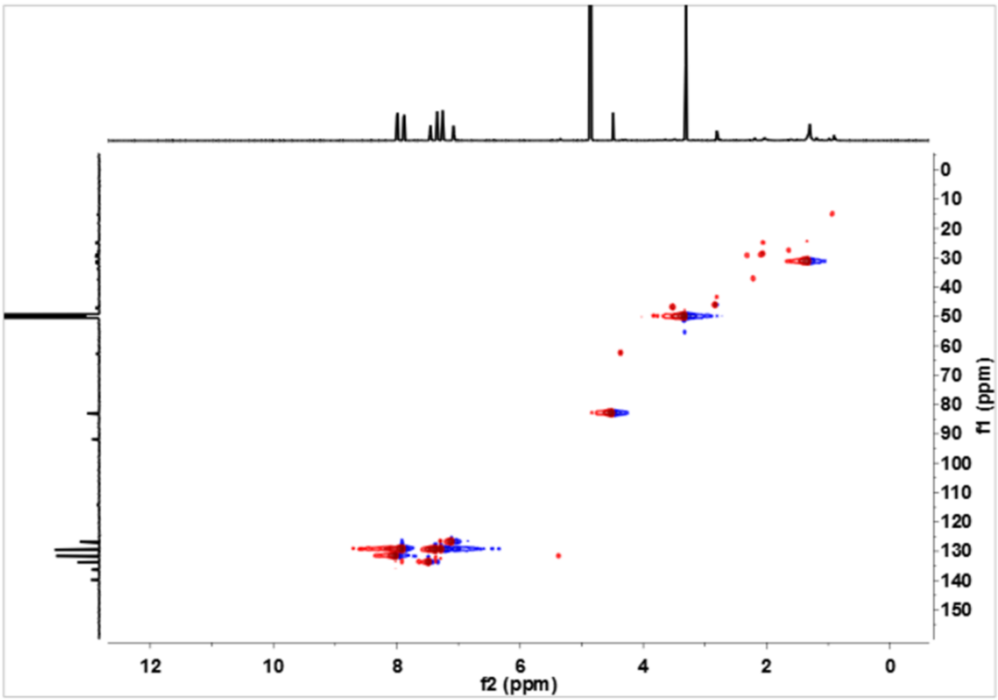

HMQC spectrum of compound 1

NMR spectrum of compound 1 (methanol)

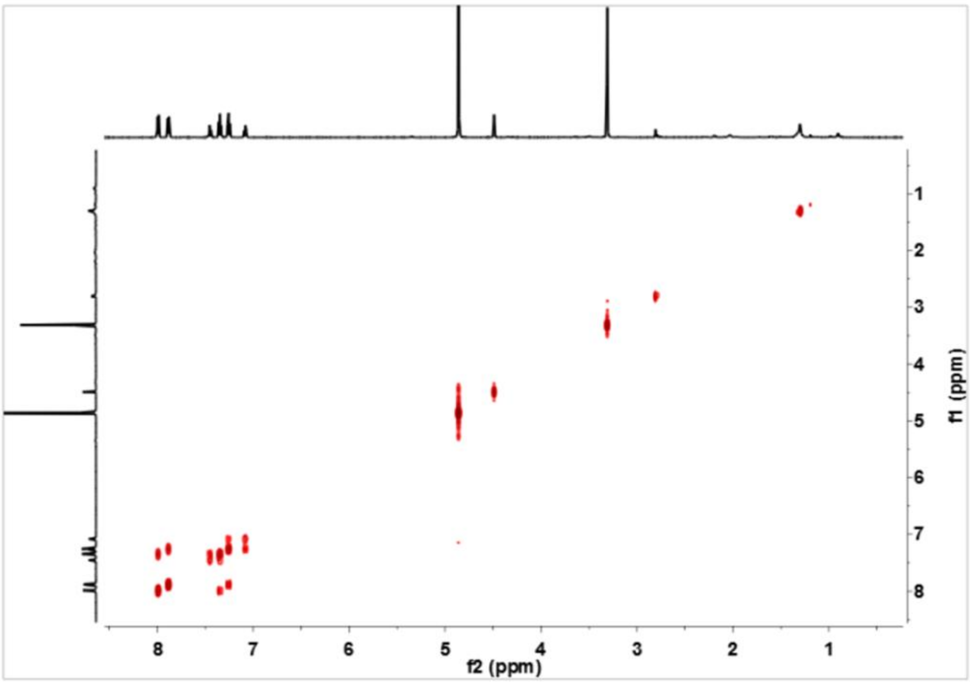

COSY spectrum of compound 1

NMR spectrum of compound 1 (methanol)

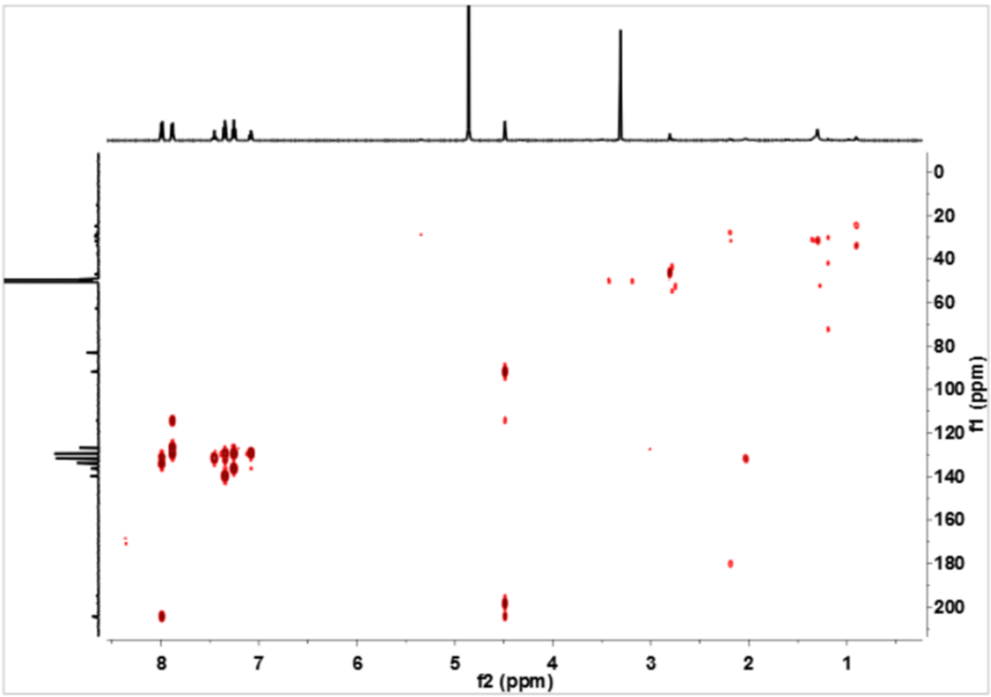

HMBC spectrum of compound 1

NMR spectrum of compound 1 (DMSO)

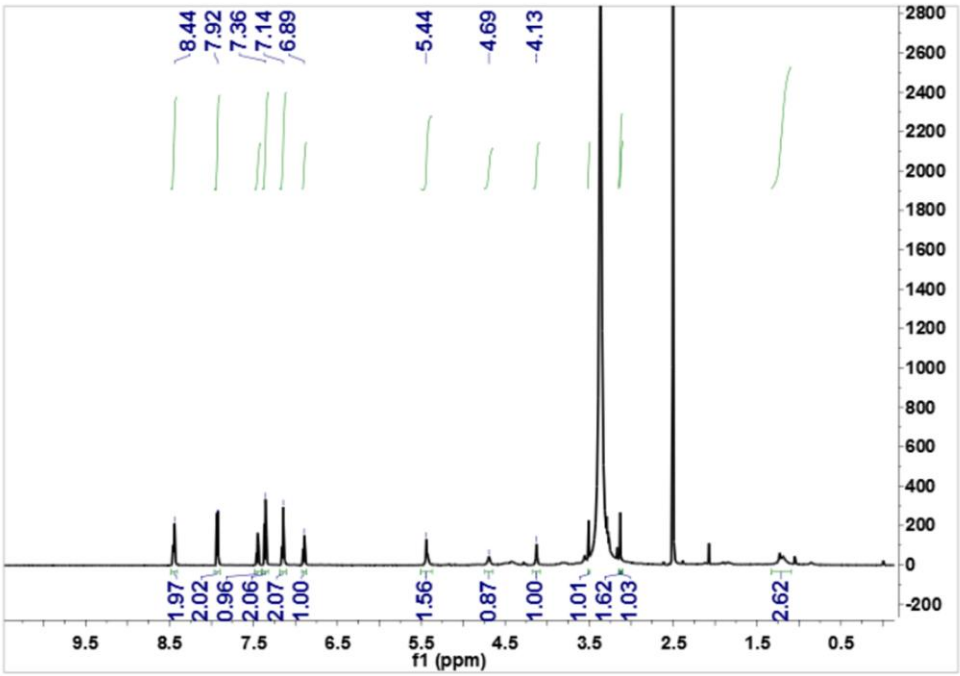

^{1}H-spectrum of compound 1

NMR spectrum of compound 1 (DMSO)

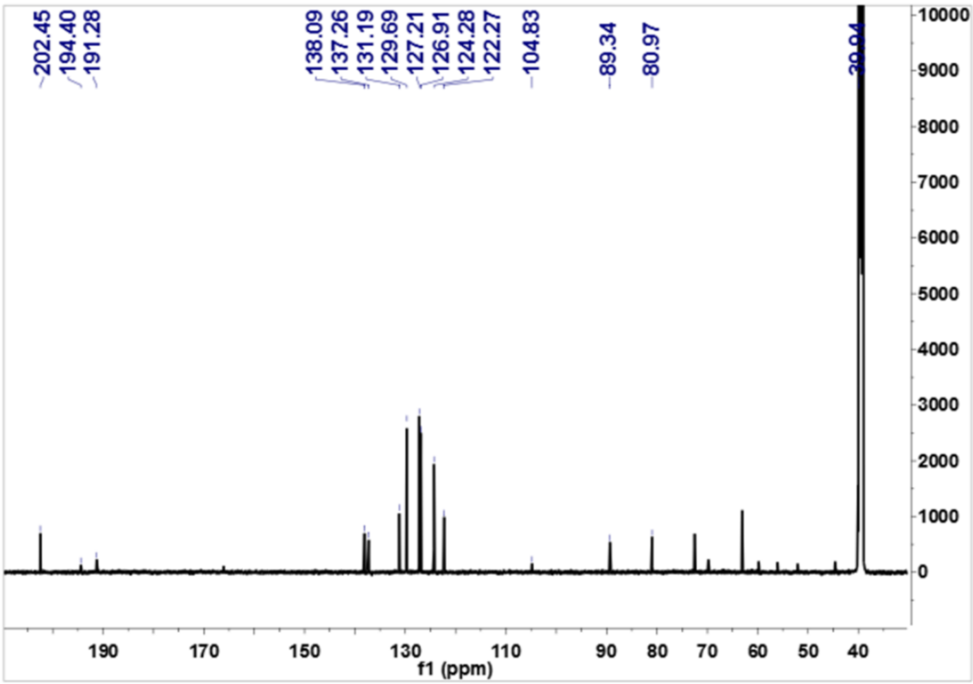

^{13}C-spectrum of compound 1

NMR spectrum of compound 1 (DMSO)

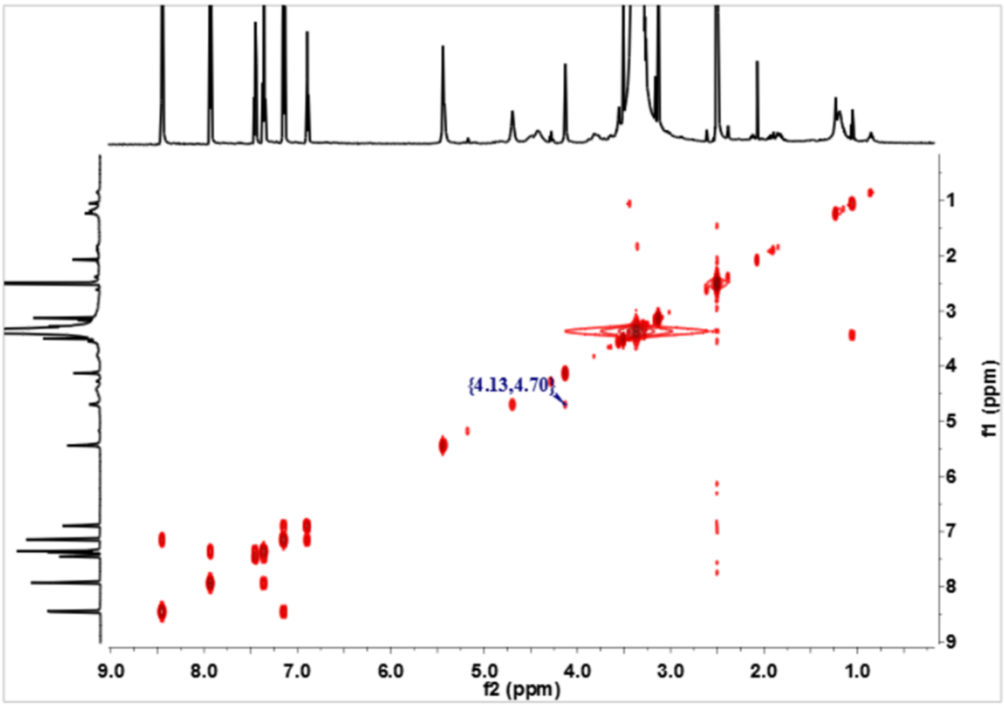

COSY spectrum of compound 1

NMR spectrum of compound 1 (DMSO)

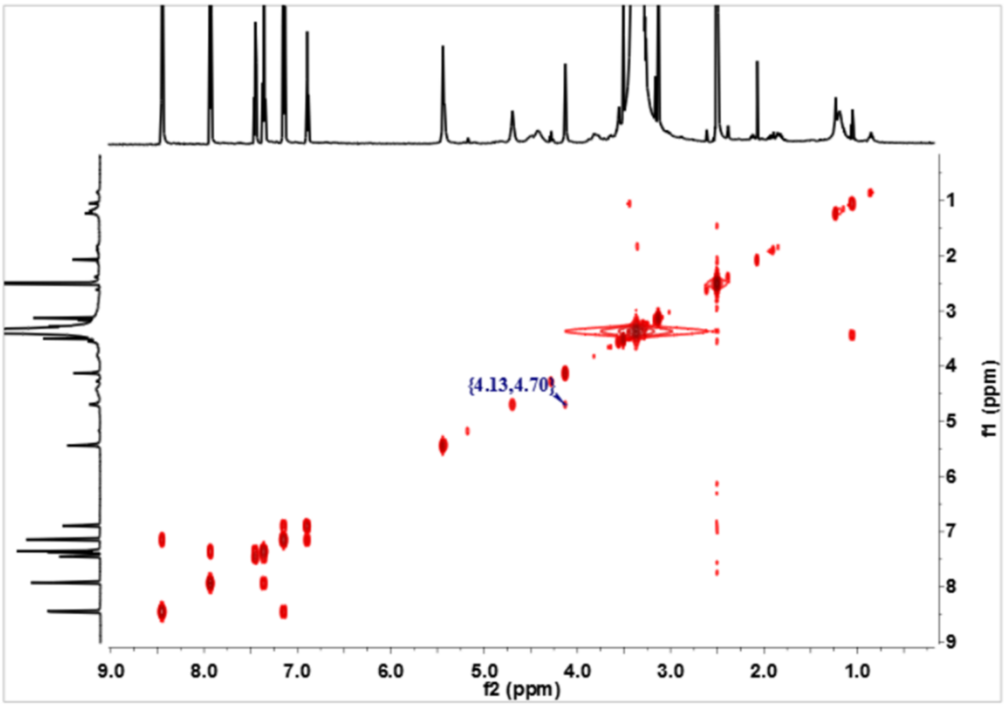

COSY spectrum of compound 1

NMR spectrum of compound 1 (DMSO)

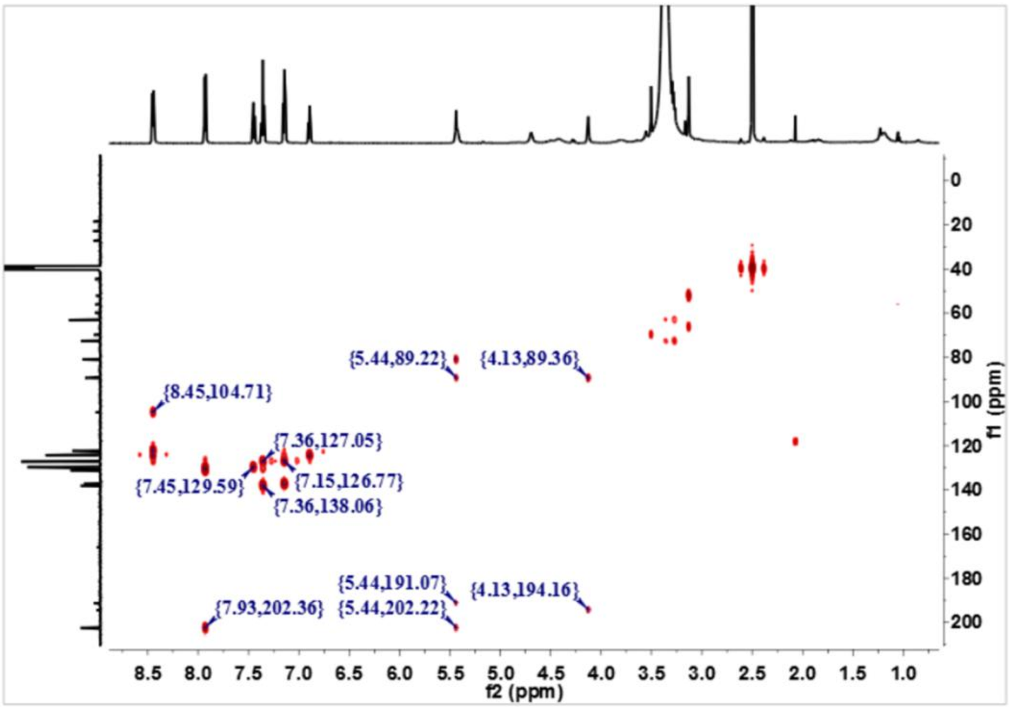

HMBC spectrum of compound 1
